# Microbiota composition of the koala (*Phascolarctos cinereus*) ocular and urogenital sites, and their association with *Chlamydia* infection and disease

**DOI:** 10.1038/s41598-017-05454-2

**Published:** 2017-07-12

**Authors:** Miranda E. Vidgen, Jonathan Hanger, Peter Timms

**Affiliations:** 1 University of the Sunshine Coast, Faculty of Science, Health, Education & Engineering, Centre for Animal Health Innovation, Locked Bag 4, Maroochydore, Qld 4558 Australia; 2 Endeavour Veterinary Ecology Pty Ltd., 1695 Pumicestone Rd., Toorbul, Qld 4510 Australia

## Abstract

Disease caused by *Chlamydia pecorum* is characterised by ocular and urogenital infections that can lead to blindness and infertility in koalas. However, koalas that are infected with *C*. *pecorum* do not always progress to disease. In other host systems, the influence of the microbiota has been implicated in either accelerating or preventing infections progressing to disease. This study investigates the contribution of koala urogenital and ocular microbiota to *Chlamydia* infection and disease in a free ranging koala population. Using univariate and multivariate analysis, it was found that reproductive status in females and sexual maturation in males, were defining features in the koala urogenital microbiota. Changes in the urogenital microbiota of koalas is correlated with infection by the common pathogen, *C*. *pecorum*. The correlation of microbiota composition and *C*. *pecorum* infection is suggestive of members of the microbiota being involved in the acceleration or prevention of infections progressing to disease. The analysis also suggests that multiple microbes are likely to be associated with this process of disease progression, rather than a single organism. While other *Chlamydia*-like organisms were also detected, they are unlikely to contribute to chlamydial disease as they are rare members of the urogenital and ocular microbiota communities.

## Introduction

Koalas (*Phascolarctos cinereus*) are a unique arboreal marsupial whose distribution spans most of eastern Australia. Populations of koalas within south-east Queensland and northern New South Wales are in population decline, due largely to urbanisation and fragmentation of the landscape resulting in reduced habitat, road trauma, dog attacks and disease^1^. The ability of individual koala populations to recover from these pressures has been limited by the pathogen *Chlamydia pecorum* ^2^. *C*. *pecorum* is the causative agent of chlamydiosis in koalas, a disease that presents as either ocular or urogenital infections that can led to blindness and infertility, and in some cases death. The presence of *C*. *pecorum* in the koala population has contributed to population declines and localised extinction due to death and reduced fecundity^2^.

The composition of individual microbiotas has been implicated in preventing, as well as, contributing to infection in some hosts systems^3–7^. Abnormalities in the human vaginal microbiota composition have been associated with susceptibility to several sexually transmitted infections (STIs) including human immunodeficiency virus (HIV)^3^, *Chlamydia trachomatis* ^4, 8^, and *Trichomonas vaginalis* ^5^. Susceptibility to STIs within the vagina can be caused by abnormal environmental conditions^8^, immunological changes^6^, and availability of essential nutrients^9^. Conversely, research has demonstrated that the normal vaginal microbiota can contribute positively to preventing transmission and acquisition of STIs, as well as, limiting disease sequelae^7^. While our understanding of the human microbiota and infection is rapidly advancing, much less is known about how animal microbiota composition influences animal infections.

Preliminary investigations of koala microbiotas have examined gastrointestinal tract, oral, and ocular sites^10, 11^. However, there has been no study of the microbial community composition of koala urogenital tracts. There have been a limited number of studies into the microbiota of marsupials. Most previous work has focussed on the gut microbiota, such as studies of faecal, foregut and/or hindgut microbiotas of koalas^10–12^, tammar wallabies^13, 14^, red kangaroos, eastern grey kangaroos, wallaroos^15^, and Tasmanian devils^16^. Other body sites that have been studied include: pouch and skin microbiota in tammar wallabies^14^ and Tasmanian devils^16^; and the oral cavity and ocular conjunctiva of koalas^11^. Only one study has investigated the marsupial urogenital tract microbiota in tammar wallabies^13^. These studies are limited to modest sample sizes with a focus on a basic understanding of the host/site microbiota. The only exception being the study of the Tasmanian devil microbiotas, which examined the influence of captivity on the microbiota^16^.

*Chlamydia*, predominantly *C*. *pecorum*, is a common and major pathogen of the koalas^2^. It remains unknown what other factors contribute to the apparent success of this pathogen in koalas; pathogen strain diversity, koala immune capability, environmental factors, or host microbiota. In addition to *C*. *pecorum*, other *Chlamydia*-like organisms (CLOs) from the broader *Chlamydiales* family have been reported in koalas^17, 18^. It is unknown how common CLOs are, what diversity exists, and if they contribute to chlamydial disease.

Due to the unique nature of *C*. *pecorum*’s koala host there is little available information on the site-specific host response to and interaction with *C*. *pecorum* infection. There is however a growing body of evidence with other *Chlamydia* species and their host that the role of co-occurring microorganisms may be vital in *Chlamydia*’s ability to establish an infection^4, 8^. In this current study we, (a) characterised for the first time, the ocular and urogenital microbiotas of male and female koalas, and (b) undertake a preliminary examination of the relationships between the microbiota profiles in *Chlamydia*-negative, *Chlamydia*-infected and *Chlamydia*-diseased koalas.

## Results and Discussion

### Taxonomic composition of koala urogenital and ocular microbiota

A total of 7,388,810 paired end sequences across all samples and controls in the library preparation were generated through MiSeq. 5,923,238 paired sequences were merged into a single read (80.16%). After primer trimming, chimeric detection, host sequence removal, and OTU assignment 3,143,739 reads were retained (53.07%). Mean reads per sample was 5,383 reads (range 26,423 to 10 reads). A total of 544 samples were included in the library preparation, with 280 samples having greater than 1000 OTUs and being included in the analysis. Based on this cut-off, only 9.8% of ocular samples were included compared to 70.5% and 82.3% of UGT and penile samples, respectively. After removal of samples with less than 1000 reads, only one sampling time point was available for the included animals. Drop-out rates for samples in this study were associated with two factors (1) low amplification rates of the 16S target due to low yields of bacterial DNA, and (2) dominance of *C*. *pecorum* reads in some samples. These technical issues were particularly prevalent in ocular samples. The dataset described in this study contains 280 microbiota samples with OTU counts greater than 1000, including 155 female UGT, 107 male penile, and 18 ocular samples.

**Figure 1:**
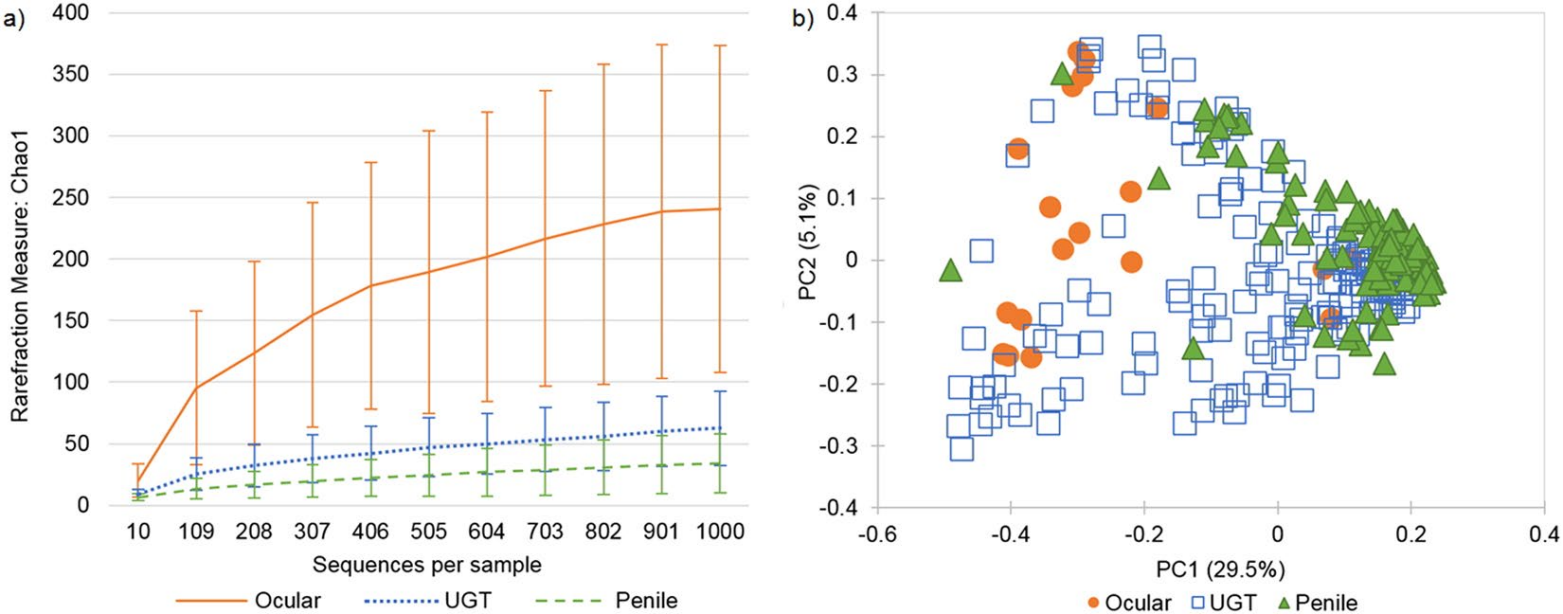
Comparison of ocular, penile and UGT microbiota from free-ranging koalas. (**a**) Phylotype richness inferred using Choa1 metric with error bars showing the standard deviation of each sample type. (**b**) PCoA of weighted UniFrac distances across all samples, as defined by sample type.

**Figure 2:**
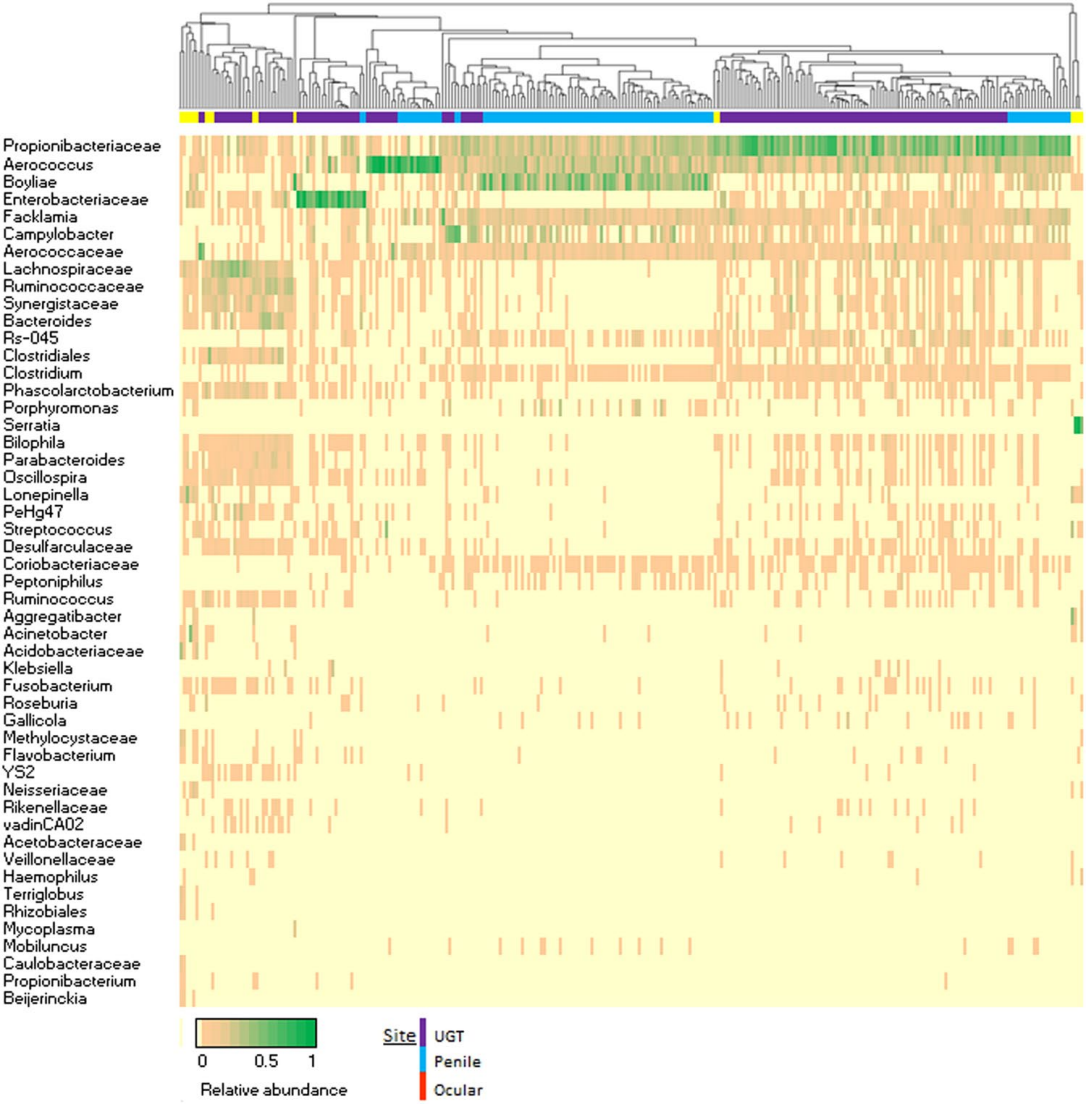
Heatmap depicting relative abundance of taxa summarised to genus level for the top 50 OTUs from UGT, penile and ocular microbiota. Relative abundance of each taxa within each sample is represent by colour on the heat map, with green indicating higher abundance. To the left of the heatmap is the nearest genus as assigned by Silva119 database and coloured based on taxonomic phylum. UPGMA dendrogram of sample relationships generated from Bray-Curtis dissimilarity matrix.

Alpha-diversity analysis (Fig. 1a) demonstrates that the koala ocular microbiota has greater OTU richness than those of the UGT and penile sites. Beta-diversity using weighted Unifrac demonstrates that the dominant OTU composition is similar between UGT and penile sites, and the ocular microbiota is more dispersed along the dominant OTU composition, but still contains many of the same OTUs (Fig. 1b). Human and animal microbiota studies have identified common sub-sets of OTUs present at multiple skin-associated sites^19, 20^. This is reflected in the koala ocular and urogenital microbiotas, which share a common sub-set of genera at these body sites (Fig. 2).

The top five most prevalent phyla amongst koala urogenital and ocular sites of this study were *Firmicutes*, *Proteobacteria*, *Actinobacteria*, *Bacteroidetes* and *Acidobacteria*. Three non-pathogenic bacteria of koalas have been formally described through traditional cultivation methods and taxonomic description; *Phascolarctobacterium faecium* ^21^, *Lonepinella koalarum* ^22^ and *Boyliae praeputiale* ^23^. All three have been detected in previous microbiome studies of koalas, with *Boyliae praeputiale* identified in these studies as *Corynebacteria* ^10, 11, 13^. Each of these microorganisms was detected within this present study.

*Firmicutes* was the most common phylum identified in the UGT samples, with the prevailing OTUs within this phyla identified as similar to members of the order *Lactobacillales* – *Aerococcus*, *Facklamia and Aerococcaceae; and the Clostridiales* - *Lachnospiraceae* and *Ruminococcaceae* (Fig. 2). *Lactobacillales* are commonly found in some vaginal microbiotas including humans and some primates^24^. However, this order is not usually a major member of the vaginal microbiota of mice^25^, ruminants^26^, guinea pigs^27^, and some arboreal primates^24^. In tammar wallabies (*Macropus eugenii*) UGT members of the order *Clostridiales* were dominant^13^, which is a significant difference from *Lactobacillales* dominant koala UGT. In tammar wallabies organisms of the genus *Lactobacillus* were not identified^13^, whereas, in koalas OTUs related to the genus *Lactobacillus* were found in the rare UGT microbiota at very low proportions.

*Actinobacteria* was the second most dominant phylum in the koala UGT. It was primarily represented by a single organism from the family *Proprionibacteriaceae* (Fig. 2). This organism was almost universally present in female UGT (n = 149/155) and male penile (n = 102/107) samples. The koala penile urethral site was similarly dominated by *Actinobacteria* and *Firmicutes*. The most common genera within the phyla Actinobacteria were organisms from the genus *Boyliae* and the family *Propionibacteriaceae* (Fig. 2). Similar to the female UGT, the penile population of Firmicutes was dominated by an *Aerococcus*, *Facklamia* and *Aerococcaceae* but did not have high levels of *Lachnospiraceae* or *Ruminococcaceae*. The human male penile microbiota is populated by organisms associated with penile epithelial mucosa and urine. This environment is dominated by a mix of microbes of the phylum *Actinobacteria* and *Firmicutes* ^28, 29^ and at the OTU level can reflect the microbiota of sexual partners^30^. The dominant genera observed in the koala penile microbiota were often observed in the koala UGT microbiota (Fig. 2), suggesting similar associations.

Vaginal microbiota of humans and other placental mammals tend to be relatively low diversity environments dominated by a *Firmicutes*, often of the order *Lactobacillales* ^24^. The main distinguishing feature of the female koala UGT microbiota, when compared to other vaginal microbiotas, was the ubiquitous presence of the *Propionibacteriaceae*. The anatomical structure of the UGT in marsupials consists of a canal that is the common site for the urethral and the three vaginal openings; the two lateral vagina and the intermittently formed pseudovaginal canal^31^. This results in a mix of urinary and genital associated organisms. Given the anatomical characteristics of the marsupial UGT, it is likely that *Proprionibacteriaceae* is part of the urinary microbiota, hence it’s near ubiquitous presence in male and female urogenital samples.

Diversity analysis shows that the ocular microbiome has a high diversity environment, and within the sample cohort is not dominated by a singular genus (Fig. 2). Our study reflects previous work on the ocular microbiota of captive koalas, which demonstrated low abundance but high diversity of microbes associated with the conjunctiva^11^, as well as a similar composition of genera to the human conjunctiva^32^. Ocular genus composition in our current study cohort of free-ranging animals has some similarities to those observed previously in captive koalas, with some consistencies in the dominant genera, namely; *Boyliae*, *Bacteroides*, *Aerococcus* and *Ruminococcacae* ^11^. However, notably absent in our study was the captive ocular study’s dominant genera *Phyllobacteriaceae* ^11^. This could be due to numerous study variations including; geographic disparity, captive versus free-ranging, and 16 S rRNA region amplified. The difference observed may also be due to individual variation between animals as this is a high diversity site that lacks universally dominant OTUs.

### Biological variables associated with koala urogenital and ocular microbiotas

Numerous studies of the penile and vaginal microbiotas in humans and other mammals have identified biological variables that are associated with the community structure and composition of the microbiota^24, 33, 34^. In both the koala penile and UGT, biological variables were correlated with differences in the microbiota. However, no variables were identified as influencing the ocular microbiota composition.

Univariate analysis of the koala ocular microbiota was limited due to sample size and lack of *C*. *pecorum*-positive samples. The analysis found no changes in diversity correlated with any of the biological variables analysed (Supplementary Table S1). It was found that when the animal was infected with *C*. *pecorum* at the ocular site, the dominant OTU observed was *C*. *pecorum* with a minimal number of non-chlamydial OTUs detected, resulting in their removal from the analysis. The main factors limiting the ocular microbiota component of this study was the depth of sequencing. Future microbiota studies on ocular *C*. *pecorum* infections in koalas will need to consider sample collection, microbe viability, and sample processing along with increasing depth of sequencing in order to obtain suitable microbiota counts associated with infection.

**Figure 3:**
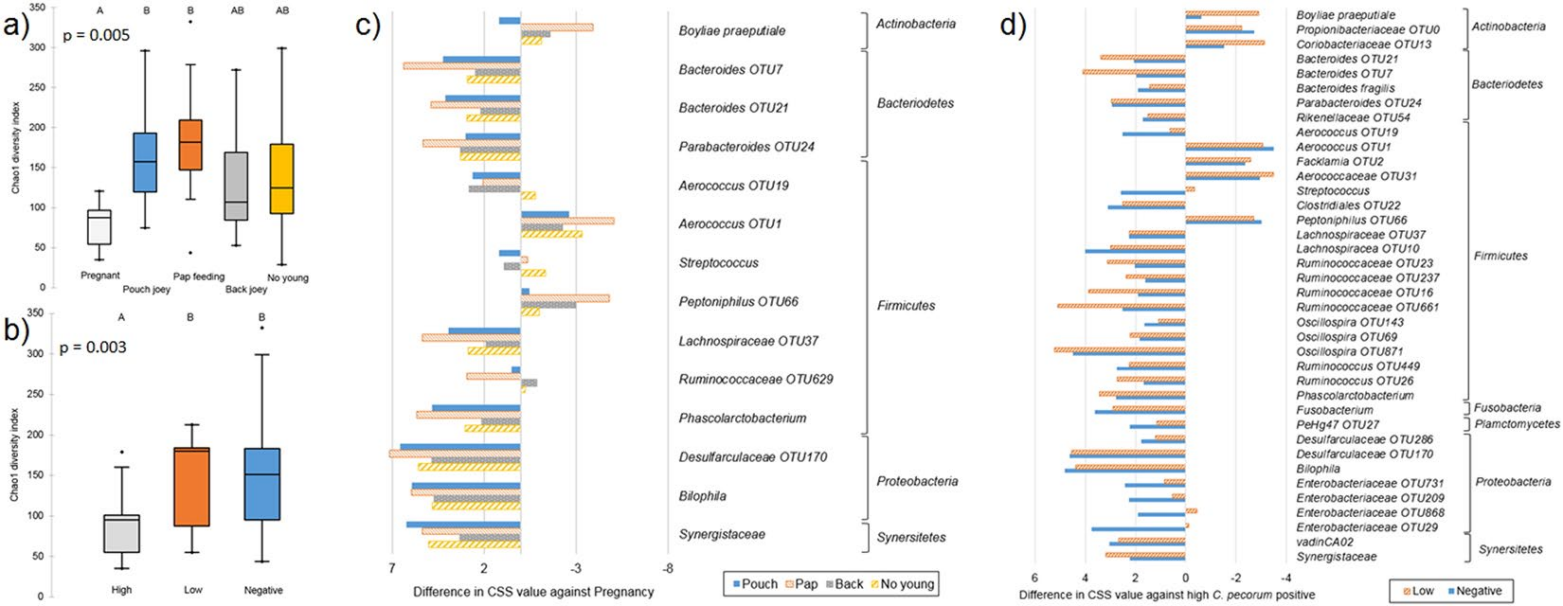
Comparative analysis of the influence of biological variables on female koala UGT microbiota for alpha-diversity and OTUs, with statistical significance (p < 0.05) as inferred by Kruskal-Wallis test. Box-plot of Chao1 diversity metric for (**a**) reproductive status, and (**b**) *C*. *pecorum* infection status-3. Bargraph of differences in OTUs values that have been normalised by CSS for (**c**) reproductive status, and (**d**) *C*. *pecorum* infection status-3.

Reproductive status, in particular pregnancy, is a dominant biological variable associated with differences in the diversity of the UGT microbiota (Fig. 3a). Comparison of alpha-diversity metrics identified significant differences for multiple metrics in reproductive status, and a single metric for disease status and season (Supplementary Table S1). This difference in reproductive status was predominantly associated with a reduction in the overall OTU richness during pregnancy, with increases in *Boyliae praeputiale* and *Peptoniphilus* OTU66, and change in the dominant *Lactobacillales*, specifically an increase in *Aerococcus* OTU1 (Fig. 3c). This change in koala UGT mirrors the microbiota changes observed during the final trimester of human pregnancy^33, 35^, which are thought to be associated with hormones influencing the microbiota^35^. A marsupial-specific hypothesis is that formation of the pseudo-vaginal canal during pregnancy temporarily introduces microbes from the upper-genital tract to the urogenital canal. When analysing reproductive status, higher OTU diversity is observed when koala joeys are present in the mother’s pouch. During this period of joey development, there are increased levels of *Bilophila*, *Desulfarculaceae* OTU170, *Lachnospiraceae* OTU37, *Ruminococcus* OTU629, *Phascolarctobacterium*, and *Bacteroides* OTU7 and OTU21 in the UGT, with *Parabacteroides* OTU24 being particularly prevalent when pouch joey is pap feeding (Fig. 3c).

**Figure 4:**
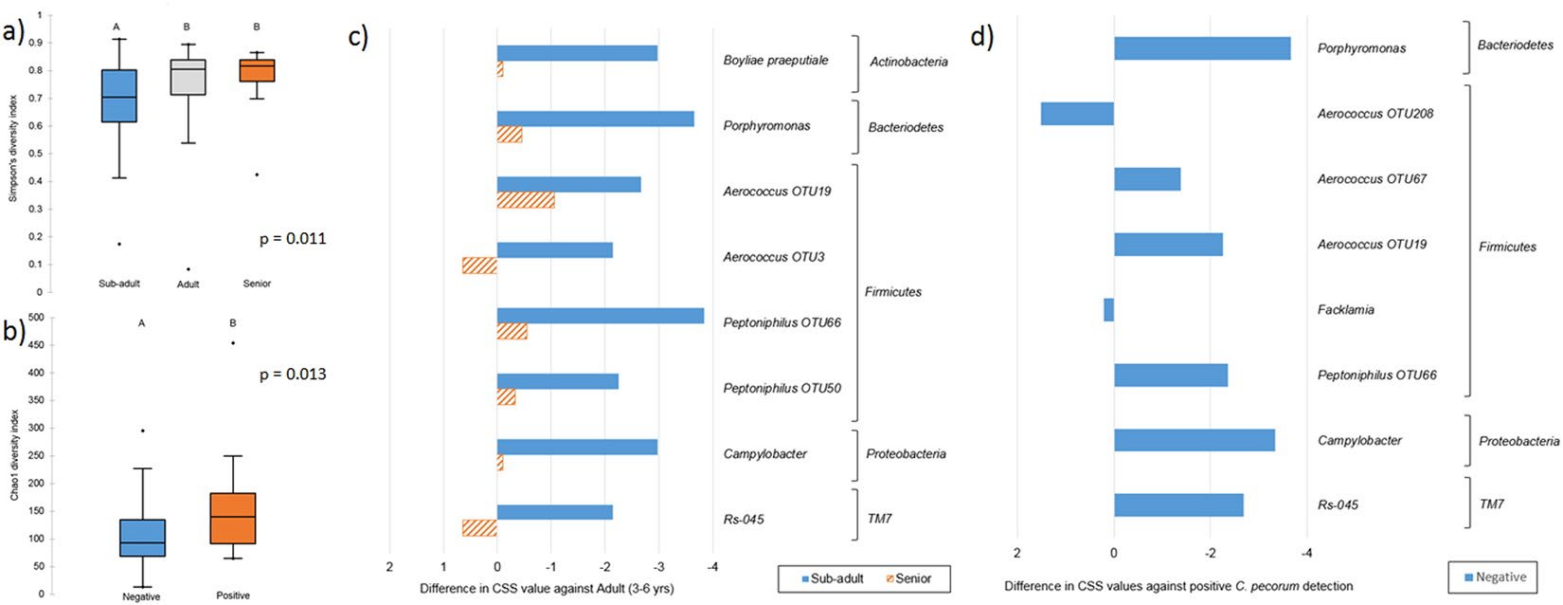
Comparative analysis of influence of biological variables on male koala penile microbiota for alpha-diversity and OTUs, with statistical significance (p < 0.05) as inferred by Kruskal-Wallis test. Box-plot of Chao1 diversity metric for (**a**) age, and (**b**) *C*. *pecorum* infection status-2. Bargraph of differences in OTUs values that have been normalised by CSS for (**c**) age, and (**d**) *C*. *pecorum* infection status-2.

Penile microbiota diversity of male koalas was influenced by age. The sub-adult animals had different alpha-diversity to adults and seniors age-group animals (Fig. 4a), with lower richness and less overall diversity. The observed changes in microbiota with sexual status is consistent with studies of the human penile microbiota of adolescent males, which shows differences in the microbiota associated with sexually experienced and inexperienced individuals^34^. Sub-adult koalas did not have any defining OTU features; rather they had lower abundance of groups such as, *Boyliae praeputiale*; *Porphyromonas* OTU9; *Aerococcus* OTU19; *Pentophillus* OTU66 and OTU50; and *Campylobacter* (Fig. 4c).

### Microbiota profiles associated with *C*. *pecorum* infection status

In addition to the biological parameters associated with differences in the microbiota profiles, *C*. *pecorum* infection status in the female UGT was associated with significant variations in microbiota composition (Fig. 3). In the UGT analysis, *C*. *pecorum* infection levels were categorised as high, low or negative (Table 1; Infection status-3). We found a specific microbiota profile associated with high *C*. *pecorum* infection, which was characterised by increased levels of *Boyliae praeputiale*, *Propionibacteriaceae* OTU0, *Coriobacteriaceae* OTU13, *Peptoniphilus* OTU66, and/or the *Lactobacillales*, *Aerococcus* OTU1, OTU2 and *Aerococcaceae* OTU31. UGTs with a low chlamydial infectious load had greater levels of *Ruminococcaceae* (OTU23, OTU237, OTU16 and OTU661) and/or *Bacteroides* (OTU21 and OTU7). The negative *C*. *pecorum* UGT correlated with more *Aerococcus* OTU19, *Streptococcus*, and/or *Enterobacteriaceae* (OTU731, OTU209, OTU868 and OTU29) (Fig. 3d). UGT samples from high *C*. *pecorum*-infected animals had lower microbiota diversity and richness, compared to those samples from animals with low level infection or negative for *C*. *pecorum* (Fig. 3b). This trend was observed in the five diversity and richness metrics tested in this study (Supplementary Table S1).

**Table 1:** Definition of variables associated with koalas used in this study.

Variable	Categories	Definition
Sample type	UGT	Sampled from the female urogenital tract
	Penile	Sampled from the male penile urethra
	Ocular	Sampled from the conjunctiva
Sex^‡^	Male	
	Female	
Age	Sub-adult	1–2 years old
	Sub-adult with young^#^	1–2 years old with young
	Adult	3–6 years old
	Senior	≥7 years old
Season^~^	Non-breeding season	March to September
	Breeding season	October to February
Reproductive status^#^	No-young	No pregnancy, dependant or independent young
	Pregnant	Pregnancy detected by ultrasound
	Pouch	Joey in pouch, aged between 0–4 months
	Pap	Joey in pouch, aged between 5–7 months
	Back	Dependant back joey
Disease status*	NAD	No disease observed at sample site
	RD	Urogenital disease detected (e.g. cystitis)
	EI	Injury to eye (i.e. cataracts)
	ED	Eye disease (i.e. chlamydial conjunctivitis)
Infection status-3	High positive	qPCR > 100 copies/µL
	Low positive	qPCR 1-100 copies/µL and >10 counts in microbiota
	Negative	No qPCR detection or <10 counts microbiota
Infection status-2	Positive	qPCR > 1copy/µL and >10 counts in microbiota
	Negative	No qPCR detection or <10 counts microbiota

^‡^Used for analysis of ocular samples only. ^#^Female UGT samples only. *Disease status at the sample site. ^~^Based on male reproductive season in South East Queensland^56^ which defines female reproductive cycles^57^.

*C*. *pecorum* infection status-3, did not correlate with changes in the penile microbiota diversity. However, analysis of the richness metrics Chao1 and PD, with infection status-2 (Fig. 4b) did yield significant differences between *C*. *pecorum* positive and negative animals (Fig. 4d). Male koalas had higher OTU richness associated with the presence of *C*. *pecorum* than those that were *Chlamydia* negative. This trend is inverse to the one observed in the female koala UGT. Penile microbiotas that were positive for *C*. *pecorum* had greater levels of *Porphyromonas* OTU9, *Aerococcus* OTU67 and OTU19, *Peptoniphilus* OTU66, Rs-045, and *Campylobacter*. Of these, *Peptoniphilus* and some *Aerococcus* OTUs were correlated to both high positive or positive *C*. *pecorum* infection in UGT and penile sites.

Univariate analysis demonstrates the complex biological influence on the koala UGT and penile microbiotas’ composition and structure. Examination of the OTUs associated with these biological or infectious states demonstrates a subset of OTUs that are contributing to differences in multiple variables. It is unclear if this infectious status correlation is dependant or independent of changes associated with variations in age or reproductive status. This raises several questions: Is the infective capability of *C*. *pecorum* influenced by the microbiota? Is there a life stage when animals are more susceptible to infection due to their microbiota? It was noted during analysis, in both diversity and the range of OTU abundance, that a *Chlamydia*-negative microbiota could have similar composition and structure to those in high *C*. *pecorum*-infected animals. This would suggest that *C*. *pecorum*-negative urogenital microbiotas can resemble positive microbiotas but in the absence of an infection event have remained negative. This provides an indication that the urogenital microbiota influences the *C*. *pecorum* infection potential, rather than *C*. *pecorum* influencing the microbiota.

### Canonical Correspondence Analysis of urogenital microbiotas

**Figure 5:**
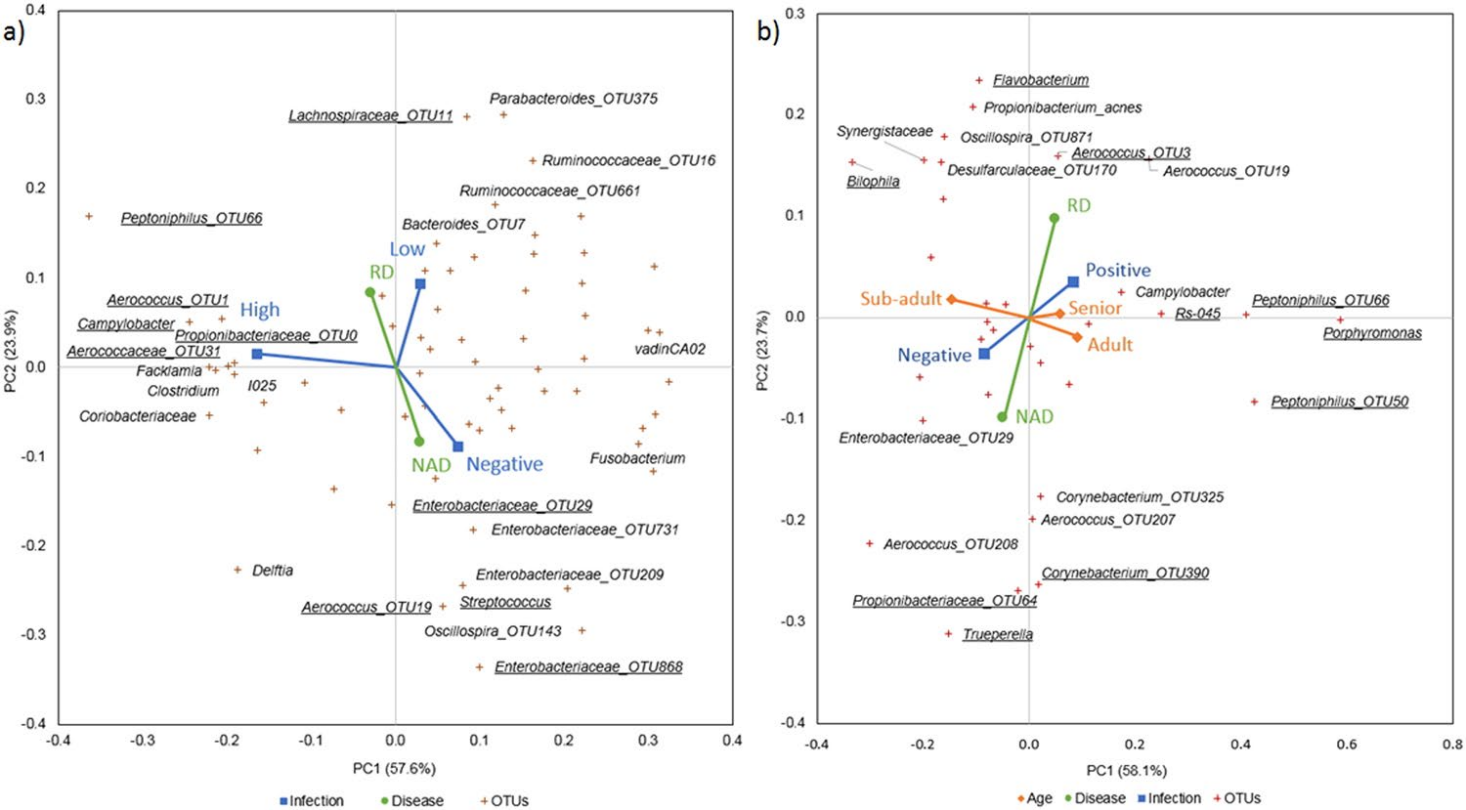
Analysis of variables associated with differences in the koala microbiota core OTUs. Canonical correspondence analysis (CCA) of (**a**) UGT (M6), and (**b**) penile (M14). Names of OTUs that contribute to ≥2.5% of variance for first two principal components (PC), PC1 or PC2 of the CCA analysis are presented with the top five contributing OTUs of each PC underlined.

To analyse the interaction between *C*. *pecorum* infection status and biological variables correlation, exploratory multivariate analysis was conducted. Canonical Correspondence Analysis (CCA) of individual variables against UGT’s core microbiota (M1 to M5), demonstrated OTUs significantly correspond for each variable tested (Table 2). When these variables were tested together, CCA identified infection status-3 and disease status to be the dominant explanatory variables for this dataset (Fig. 5a). The primary principal component demonstrated that high *C*. *pecorum* infection status associates with a different OTU composition compared to low and negative samples. The top 5 contributing OTUs were; *Aerococcus* OTU1, *Aerococcus* OTU31, *Peptoniphilus* OTU66, *Propionibacteriaceae* OTU0, and *Campylobacter* (Supplementary Table S2). It is in the secondary principal component that differences between low and negative C. pecorum detections are observed. These variations interestingly co-correlated with disease status, with low *C*. *pecorum* detection being correlated with reproductive disease (RD) and negative *C*. *pecorum* infection status with no associated disease (NAD). The top 5 contributing OTUs associated with low *C*. *pecorum* were*; Lachnospiraceae* OTU11, *Aerococcus* OTU19, *Streptococcus*, *Enterobacteriaceae* OTU868 and *Enterobacteriaceae* OTU29 (Supplementary Table S2).

**Table 2:** Models for Canonical Correspondence Analysis of microbiota OTUs for UGT and penile sites.

Source	CCA model variables	Pseudo F	p-value	Model	Fig.
UGT	Infection-3	1.466	0.0001****	M1	
	Disease	0.825	0.028*	M2	
	Reproductive-status	0.564	0.053^•^	M3	
	Season	0.727	0.054^•^	M4	
	Age	0.544	0.1^•^	M5	
	Infection-3 + Disease	1.249	0.0001****	M6	5a
	Infection-3 + Disease + Season + RS	0.814	0.0001****	M7	
	Infection-3 + Disease + Season + RS + Age	0.781	0.0001****	M8	
Penile	Age	0.6	0.005**	M9	
	Disease	0.54	0.045*	M10	
	Infection-2	0.505	0.049*	M11	
	Season	0.361	0.238	M12	
	Age + Disease	0.578	0.004**	M13	
	Age + Disease + Infection-2	0.532	0.004**	M14	5b

p-value: ^•^0.1–0.05, *<0.05, **<0.01, ***<0.001, ****<0.0001.

CCA of the penile microbiota identified age and disease status as the dominant explanatory variables, with infection status a co-correlated variable (M14) (Fig. 5b). The primary principal component is associated with age, with sub-adults being different in OTU composition to adult and senior animals (Fig. 5b), which are closely associated in both the primary and secondary principal components. The secondary principal component is dominated by disease status, with positive infection status co-correlating with RD, adult age, and senior age, and negative infection status correlating with NAD and sub-adult age. The dominant variable of the third principal component was infection status, with the OTUs that correlated with a positive infection status being, *Aerococcus* OTU67, *Flavobacterium*, *Propionibacteriaceae* OTU64, *Porphyromonas*, and *Desulfarculaceae* OTU170 (Supplementary Table S3).

The study highlights a few organisms of interest. However, the differences observed do not represent patterns of presence or absence but rather mean abundance of an OTU within the biological variable cohorts. This suggests that there is not a direct correlation between *C*. *pecorum* and presence or absence of a single member of the microbiota, but rather a correlation in the overall microbiota composition. This is not an unexpected result for a urogenital chlamydial infection. In the case of *C*. *trachomatis* infections of the human vaginal tract, the presence of indole-producing organisms support chlamydial replication by preventing interferon-γ mediated starvation of tryptophan^4^ and differences in the dominant *Lactobacillus* influence infectivity by altering the environment and glucose metabolism^8^. In both of these examples, it is not the organism *per se* that is important, but rather the metabolic resources and environmental influence of the organism, or multiple organisms, that affect the infective potential of *C*. *trachomatis*.

### Chlamydial composition of koala urogenital and ocular microbiotas

**Figure 6:**
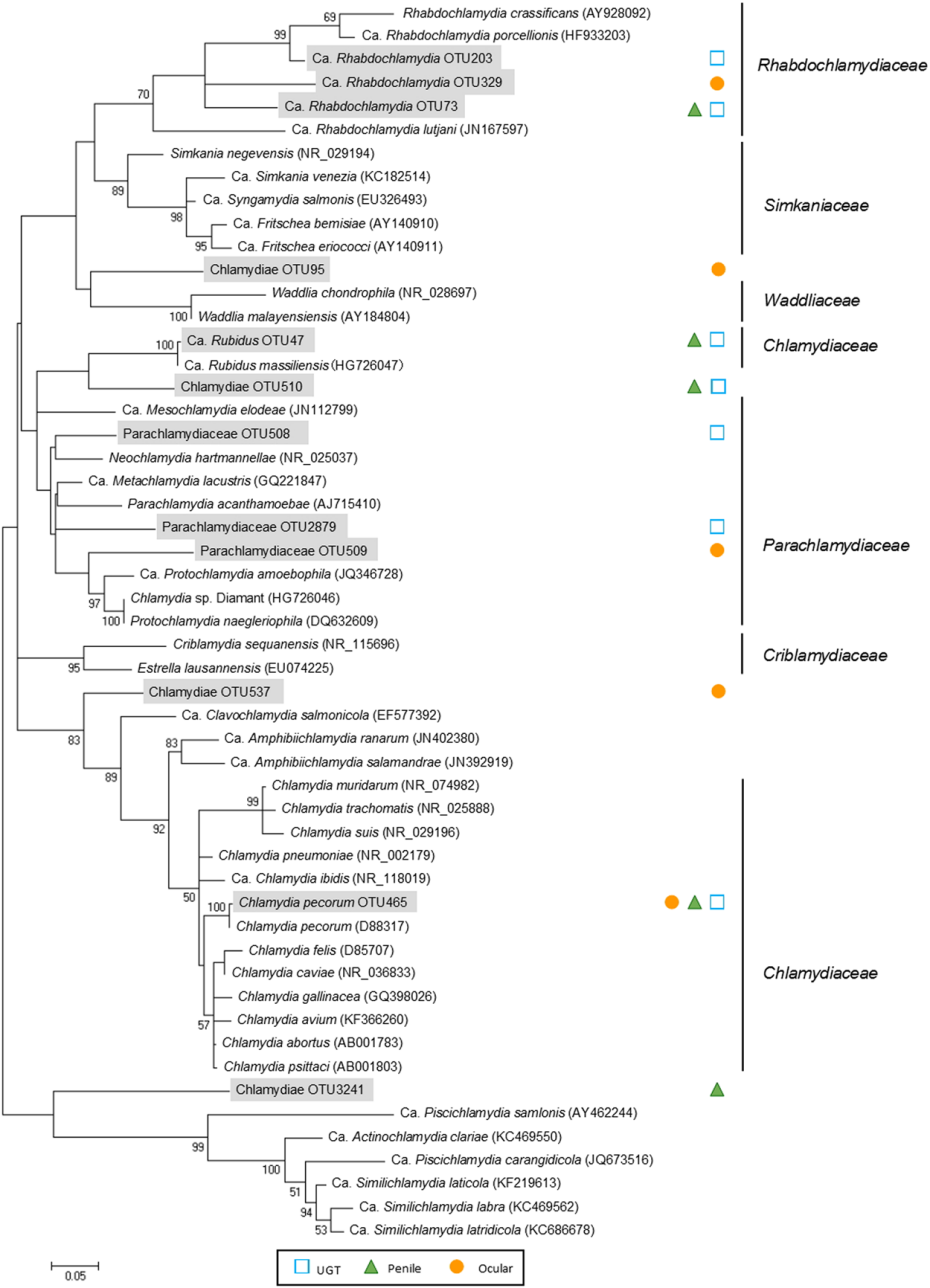
16S rRNA maximum likelihood tree of known *Chlamydiae* and undescribed *Chlamydiae* OTUs from the koala microbiota. Percentage for bootstrap re-sampling (n = 1000) given for branches with greater than 50% occurrence during re-sampling. OTUs highlighted in grey were identified in this study. Bar represents 0.05 nucleotide substitutions.

The target *Chlamydia* species of this study is *C*. *pecorum*, however *C*. *pneumoniae* has previously been identified in koalas^36^ and *C*. *psittaci* is endemic to birds of the study region^37^. Within the genus *Chlamydia*, *C*. *pecorum* was the only species that we identified in the microbiota of these samples (Fig. 6). In previous studies of koalas, the presence of chlamydial organisms from outside the *Chlamydiaceae* family, often known as *Chlamydia*-like organisms (CLOs), have been identified^17, 18^. We therefore examined our microbiota data for the presence of any such CLOs. Within our total microbiota dataset (n = 280), we identified 12 rare OTUs in 14 samples as *Chlamydiae*, which were phylogenetically distinct from *C*. *pecorum* and the genus *Chlamydia* (Fig. 6). Two OTUs were closely related to Candidatus *Rubidus massiliensis* (>99% similarity) and *Ca*. *Rhabdochlamydia porcellionis* (95–97% similarity). Both of these characterised organisms belong to a group of environmental *Chlamydiae* ^38, 39^. Six OTUs clustered within two existing families; *Parachlamydiaceae* (OTU508, OTU509 and OTU2879) and *Rhabdochlamydiaceae* (OTU203, OTU329 and OTU73). The remaining four OTUs (*Chlamydiae* OTU95, OTU510, OTU537 and OTU3241) were not associated with an existing *Chlamydiae* family and have a 90 to 97% BLAST similarity to uncultured microbes previously detected in environmental samples (Supplementary Table S4).

These rare CLOs were found either as the single *Chlamydiae* inhabitant of a site (n = 9) or as co-habitants of sites with *C*. *pecorum* and other *Chlamydiae* (n = 5). Cohabitation of *C*. *pecorum* and CLOs has previously been reported^17^. However, in our study it was rare to find CLOs present during *C*. *pecorum* infections of the urogenital tracts of male and female koalas (n = 2/53), with it slightly more common to observe co-habitation of CLOs and *C*. *pecorum* in ocular sites (n = 2/9). Ocular sites have a higher prevalence of rare CLOs with 15% of samples having a *Chlamydiae* OTU. This is compared with penile and UGT samples in which Chlamydiae OTUs were identified in only 3.7% and 4.5% of samples, respectively. Comparison of the OTUs between sample types suggests that the rare ocular *Chlamydiae* microbiota is different to the organisms observed in the urogenital tract. In the penile and UGT of koalas, the rare *Chlamydiae* microbiota is shared, with three OTUs identified in the urogenital tracts of both sexes and a further four OTUs identified in the UGT of a single sex (female n = 3, male n = 1). The phylogenetic lineage of these novel *Chlamydiae* did not correlate with any particular body site.

## Conclusion

This study represents the first examination of the koala urogenital tract microbiota. Our preliminary results indicate that the koala UGT microbiota is dominated by *Aerococcus*-like organisms that are from the order *Lactobacillales* with *Lactobacillus*-like OTUs being part of their rare microbiota. The size of this study has enabled us to go beyond a basic understanding of the community structure for the koala urogenital microbiota and examine the correlation of biological variables with microbiota’s structure. For females, reproductive stage, specifically pregnancy, is the dominant feature influencing diversity. The male penile microbiota is affected by sexual maturation and sexual experience. Outside of the biological variables, we found a correlation of microbiota composition with *C*. *pecorum* infection identified in both urogenital sites. The correlation of microbiota composition and *C*. *pecorum* infection is suggestive of members of the microbiota being involved in the acceleration or prevention of infections progressing to disease. However, further work is needed to (1) determine the organisms that are involved in this process, and (2) if this process is dependant or independent of changes associated with variations in age or reproductive status. To do this large samples size will be needed and examination of animals at multiple time points.

The koala ocular microbiota is of low abundance but high diversity. The ocular site lacks a single dominant organism with broad individual animal diversity. The ability to study *C*. *pecorum* infections was limited by the infective agents dominating detection over non-*C*. *pecorum* reads. Moving forward it would be necessary to maximise depth of sequencing to ensure that the underlying microbiota is adequately detected.

Although rare CLOs were observed, in this study *C*. *pecorum* was the dominant chlamydial organism in the ocular and urogenital microbiotas. The rarity and low prevalence of CLOs would suggest that they are part of the commensal microbiota, and there is no evidence to suggest that other CLOs are contributing to chlamydial disease.

The koala urogenital microbiomes are dynamic with changes in composition that are associated with life stage. This study provides preliminary evidence that *C*. *pecorum* infection and its progression to disease may be influenced by other microorganisms present in the urogenital tract of koalas. This highlights the importance of research that incorporates a whole system approach to the investigation of Chlamydial disease in these iconic marsupials.

## Methods

### Sample collection

Animals included in the study (n = 232) were part of a larger population-wide study by the Queensland Government Department of Transport and Main Roads (as part of the Moreton Bay Rail Link project), conducted between 2012 and 2015 in the Moreton Bay Region, Queensland, Australia. Samples were collected from ocular (both sexes), urogenital tract (UGT) of females and penile site of males, as described previously^40^. In total 544 samples (ocular and/or urogenital) from 232 animals were included in the study with sampling at 1 or 2 time points (Supplementary Table S5). All procedures were approved by the University of the Sunshine Coast Animal Ethics Committee (Animal ethics number AN/A/13/80) and by the Queensland Government (Scientific Purposes Permit, WISP11532912). All experiments were performed in accordance with relevant guidelines and regulations.

### DNA extraction and *Chlamydia pecorum* detection with qPCR

Genomic DNA (DNA) was extracted from koala urogenital and ocular swabs using QIAamp DNA minikit (Qiagen, Valencia, CA) as previously described^41^. The extracted DNA was screened for the presence of *C*. *pecorum* using a species-specific quantitative real-time PCR (qPCR) that targeted the 16 S rRNA gene^41^ (Supplementary Table S6). The reaction mix contained: 5–50 ng DNA; 1x QuantiTect SYBR Green PCR Master Mix (Qiagen, Valencia, CA); and 10 µM of each primer made up to a final volume of 25 µL with molecular grade water. Thermocycling was performed on a RotorGeneQ 5-plex HRM platform (Qiagen, Valencia, CA) using a 15-minute initial denaturation at 94 °C followed by 40 cycles at: 94 °C for 15 seconds; 57 °C for 30 seconds; 72 °C for 25 seconds for amplification.

### Illumina library preparation and sequencing

A library was prepared for the V3-V4 region of the 16S rRNA gene using dual-indexed primers^42^ (Supplementary Table S6). Each reaction plate contained a positive and negative control. PCR amplicons were generated in duplicate using barcoded 319F and 806R primers with reaction mix containing: 5–50 ng DNA; 1 x HiFi Hot Start Ready Mix (KAPA Biosystems; Cape Town, South Africa); and 1 µM of each primer made up to a final volume of 25 µL with molecular-grade water. Thermocycling was performed on a Nexus Mastercycler (Eppendorf; Hamburg, Germany) using a 3 minute initial denaturation at 95 °C followed by 30 cycles at: 95 °C for 15 seconds; 55 °C for 15 seconds; 72 °C for 30 seconds, and then a final 72 °C extension for 5 mins. PCR products were cleaned and standardised using SequalPrep Normalisation kit (Invitrogen; Carlsbad, CA) and then pooled. The pooled library preparation was concentrated using DNA Clean and Concentrator-25 (Zymo Research; Irvine, CA), and residual primers removed using a Gel DNA Recovery Kit (Zymo Research; Irvine, CA). The pooled library preparation was sequenced on MiSeq platform using 600-cycle kit chemistry v3 (Illumina; San Diego, CA) by Ramaciotti Centre for Genomics (Sydney, Australia).

### Data processing

The raw data files were pre-processed using the method previously described^42^, with PEAR for merging the paired end reads^43^, CutAdapt for trimming barcode/primer^44^, and vsearch for detection and removal of chimeric sequences^45^. Pre-processed sequence data were then analysed using QIIME (v1.9)^46^. Operational taxonomic units (OTUs) were picked using an open-reference strategy^47^ with pre-filtering for non-bacterial amplicons. At a similarity cut-off of 97%, OTUs were identified initially using SortMeRNA^48^ and SUMACLUST^49^ with secondary OTU picking utilising uclust^50^ and PyNAST^51^. During both stages taxonomy was assigned using the Silva 119 database^52^. Chloroplast and OTU singletons were removed from the dataset. To avoid biasing during subsequent statistical analysis, *C*. *pecorum* OTUs were removed from the OTU table and *C*. *pecorum* microbiota counts were used in assessing infection status (Table 1). The highest count for a single OTU within the negative control samples was eight. As such, OTUs with a prevalence below 10 counts were removed from the OTU table. After processing, samples with less than 1000 counts/samples were removed from analysis. For proceeding analyses the data was formatted into three different types; (1) abundance data (raw data), (2) relative abundance at the taxonomic level of genus, and (3) cumulative sum of squares (CSS) normalisation of abundance data^53^.

### Sequence diversity analysis

Microbial diversity was evaluated within samples (α-diversity) and between samples (β-diversity) using QIIME and the abundance data set. Alpha diversity of the sample types was assessed using alpha rarefaction of Chao1 richness estimator (Chao1), whole-tree phylogenetic diversity metric (PD), and OTU counts at 10 increments between 10 and 1000 counts/samples. Beta diversity was evaluated using weighted and unweighted UniFrac^54^ with rarefaction to 1000 counts/sample. Results of the weighted UniFrac analysis are presented as Principal Coordinate Analysis (PCoA) of the first two principal components. Phylogenetic diversity was depicted by heat map-based analysis using relative abundance data summarised to the taxonomic level of genus. The relationship between samples was represented with the heatmap with an unweighted pair group method with arithmetic mean (UPGMA) dendrogram generated from a Bray-Curtis dissimilarity matrix.

### Statistical analysis

Univariate analysis of the samples was conducted to identify factors that influence the urogenital and ocular microbiota of male and female koalas. Variables associated with animal and disease status were categorised based on data collected during the Moreton Bay Rail Link project (Table 1). The categorisation of *C*. *pecorum* infection status was based on qPCR testing, and *C*. *pecorum* detection in the microbiota analysis. *C*. *pecorum* results were examined using two variables groupings; Infection status-2, and Infection status-3 (Table 1).

Univariate analysis was conduct using the α-diversity metrics testing for richness and diversity; Chao1, Abundance-based Coverage Estimator (ACE), PD, Shannon-Wiener diversity index (Shannon), and Simpson’s index (Simpson). Calculation of the diversity measure was conducted using abundance data. Samples were defined by each associated variable category (Table 1) and diversity metrics for a category were compared within variables using Kruskal-Wallis test using Dunn’s procedure with a Bonferroni’s correction calculated (XLStat; New York, NY) (Supplementary Table S1).

Canonical correspondence analysis (CCA) of the OTUs was performed using CSS normalised microbiota data that contained the OTUs present in >25% samples microbiota (Supplementary Table S7). Each variable was tested individually against the OTU datasets using permutation test (n = 1000) for the CCA. Those individual variables with p-value < 0.1 were combined in stepwise from lowest to highest in iterative CCA.

### Phylogenetic analysis

The 16S rRNA gene sequences (~429 bp) identified as belonging to the phylum *Chlamydiae* during OTU picking were aligned using Silva^52^. Nearest neighbour was identified using BLAST search (Supplementary Table S4). Maximum Likelihood analysis with bootstrapping (n = 1000) was performed with MEGA7^55^.

### Data availability

Sequences files are available at the NCBI Sequence Read Archive, accession number SRP092369.

## Electronic supplementary material

Supplementary Tables S1 to S7
