# Supplementary material for: Microbiota composition of the koala (Phascolarctos cinereus) ocular and urogenital sites, and their association with Chlamydia infection and disease
Source: Sci Rep. 2017 Jul 12;7:5239. doi: 10.1038/s41598-017-05454-2 (PMC5507983; doi:10.1038/s41598-017-05454-2)
Supplement: Supplementary file 1 — Supplementary Tables S1 to S7 [file 41598_2017_5454_MOESM1_ESM.pdf]

# Microbiota composition of the koala (*Phascolarctos cinereus*) ocular and urogenital sites, and their association with *Chlamydia* infection and disease.

Miranda E. Vidgen[1], Jonathan Hanger[2] and Peter Timms[1*]

[1] University of the Sunshine Coast, Faculty of Science, Health, Education & Engineering, Centre for Animal Health Innovation, Locked Bag 4, Maroochydore, Qld 4558, Australia

[2] Endeavour Veterinary Ecology Pty Ltd, 1695 Pumicestone Rd, Toorbul, Qld 4510, Australia

**Corresponding Author:**

Peter Timms

Postal Address: University of the Sunshine Coast, Locked Bag 4, Maroochydore, Qld 4558, Australia

Email address: ptimms@usc.edu.au

**Table S1: Kruskall-Wallis results for alpha-diversity metrics against biological variables of urogenital and ocular microbiome**

| Sample Type | **Variable** | **Alpha-diverity metric** | **p-value** | **Bonferroni corrected significance level** | **Significance** |
|---|---|---|---|---|---|
| UGT | Age_group | Chao1 | 0.211 | 0.0083 | NSD |
| UGT | Age_group | PD | 0.132 | 0.0083 | NSD |
| UGT | Age_group | Shannon | 0.029 | 0.0083 | NSD |
| UGT | Age_group | Simpson | 0.052 | 0.0083 | NSD |
| UGT | Age_group | ACE | 0.442 | 0.0083 | NSD |
| UGT | Disease_status | Chao1 | 0.142 | 0.05 | NSD |
| UGT | Disease_status | PD | 0.019* | 0.005 | SD |
| UGT | Disease_status | Shannon | 0.217 | 0.05 | NSD |
| UGT | Disease_status | Simpson | 0.356 | 0.05 | NSD |
| UGT | Disease_status | ACE | 0.056 | 0.05 | NSD |
| UGT | Reproductive_status | Chao1 | 0.005** | 0.005 | SD |
| UGT | Reproductive_status | PD | 0.004** | 0.005 | SD |
| UGT | Reproductive_status | Shannon | 0.053 | 0.005 | NSD |
| UGT | Reproductive_status | Simpson | 0.052 | 0.005 | NSD |
| UGT | Reproductive_status | ACE | 0.003** | 0.005 | SD |
| UGT | Season | Chao1 | 0.246 | 0.05 | NSD |
| UGT | Season | PD | 0.021* | 0.05 | SD |
| UGT | Season | Shannon | 0.694 | 0.05 | NSD |
| UGT | Season | Simpson | 0.503 | 0.05 | NSD |
| UGT | Season | ACE | 0.026* | 0.05 | SD |
| UGT | Infection_status-3 | Chao1 | 0.003** | 0.0167 | SD |
| UGT | Infection_status-3 | PD | 0.001*** | 0.0167 | SD |
| UGT | Infection_status-3 | Shannon | 0.0001**** | 0.0167 | SD |
| UGT | Infection_status-3 | Simpson | 0.005** | 0.0167 | SD |
| UGT | Infection_status-3 | ACE | 0.012* | 0.0167 | SD |
| Penile | Age_group | Chao1 | 0.365 | 0.0167 | NSD |
| Penile | Age_group | PD | 0.467 | 0.0167 | NSD |
| Penile | Age_group | Shannon | 0.033 | 0.0167 | NSD |
| Penile | Age_group | Simpson | 0.011* | 0.0167 | SD |
| Penile | Age_group | ACE | 0.276 | 0.0167 | NSD |
| Penile | Disease_status | Chao1 | 0.054 | 0.05 | NSD |
| Penile | Disease_status | PD | 0.074 | 0.05 | NSD |
| Penile | Disease_status | Shannon | 0.702 | 0.05 | NSD |
| Penile | Disease_status | Simpson | 0.814 | 0.05 | NSD |
| Penile | Disease_status | ACE | 0.043* | 0.05 | SD |
| Penile | Season | Chao1 | 0.484 | 0.05 | NSD |
| Penile | Season | PD | 0.667 | 0.05 | NSD |
| Penile | Season | Shannon | 0.896 | 0.05 | NSD |
| Penile | Season | Simpson | 0.896 | 0.05 | NSD |
| Penile | Season | ACE | 0.261 | 0.05 | NSD |
| Penile | Infection_status-2 | Chao1 | 0.027* | 0.05 | SD |
| Penile | Infection_status-2 | PD | 0.068 | 0.05 | NSD |
| Penile | Infection_status-2 | Shannon | 0.088 | 0.05 | NSD |
| Penile | Infection_status-2 | Simpson | 0.191 | 0.05 | NSD |
| Penile | Infection_status-2 | ACE | 0.013* | 0.05 | SD |
| Penile | Infection_status-3 | Chao1 | 0.061 | 0.0167 | NSD |
| Penile | Infection_status-3 | PD | 0.163 | 0.0167 | NSD |
| Penile | Infection_status-3 | Shannon | 0.163 | 0.0167 | NSD |

| | | | | | |
|---|---|---|---|---|---|
| Penile | Infection_status-3 | Simpson | 0.168 | 0.0167 | NSD |
| Penile | Infection_status-3 | ACE | 0.034 | 0.0167 | NSD |
| Ocular | Age_group | Chao1 | 0.313 | 0.0083 | NSD |
| Ocular | Age_group | PD | 0.359 | 0.0083 | NSD |
| Ocular | Age_group | Shannon | 0.92 | 0.0083 | NSD |
| Ocular | Age_group | Simpson | 0.901 | 0.0083 | NSD |
| Ocular | Age_group | ACE | 0.151 | 0.0083 | NSD |
| Ocular | Sex | Chao1 | 0.509 | 0.05 | NSD |
| Ocular | Sex | PD | 0.563 | 0.05 | NSD |
| Ocular | Sex | Shannon | 0.869 | 0.05 | NSD |
| Ocular | Sex | Simpson | 0.934 | 0.05 | NSD |
| Ocular | Sex | ACE | 0.62 | 0.05 | NSD |
| Ocular | Disease_status | Chao1 | 0.655 | 0.05 | NSD |
| Ocular | Disease_status | PD | 0.576 | 0.05 | NSD |
| Ocular | Disease_status | Shannon | 0.219 | 0.05 | NSD |
| Ocular | Disease_status | Simpson | 0.371 | 0.05 | NSD |
| Ocular | Disease_status | ACE | 0.737 | 0.05 | NSD |
| Ocular | Season | Chao1 | 0.509 | 0.05 | NSD |
| Ocular | Season | PD | 0.62 | 0.05 | NSD |
| Ocular | Season | Shannon | 0.248 | 0.05 | NSD |
| Ocular | Season | Simpson | 0.215 | 0.05 | NSD |
| Ocular | Season | ACE | 0.62 | 0.05 | NSD |
| Ocular | Infection_status-2 | Chao1 | 0.293 | 0.05 | NSD |
| Ocular | Infection_status-2 | PD | 0.483 | 0.05 | NSD |
| Ocular | Infection_status-2 | Shannon | 1 | 0.05 | NSD |
| Ocular | Infection_status-2 | Simpson | 0.661 | 0.05 | NSD |
| Ocular | Infection_status-2 | ACE | 0.188 | 0.05 | NSD |

p-value: * <0.05, ** <0.01, *** <0.001, ***<0.0001. NSD, no significant difference; SD, significant difference.

**Table S2: Percentage contribution of core OTUs to the canonical correspondence analysis of UGT microbiome with infection-3 and disease as variables (M6).**

| OTUs | Contribution to CCA 1 |
|---|---|
| Aerococcaceae_OTU31 | 6.45% |
| Aerococcus_OTU1 | 6.39% |
| Propionibacteriaceae_OTU0 | 6.25% |
| Peptoniphilus_OTU66 | 5.92% |
| Campylobacter | 5.91% |
| Facklamia | 5.34% |
| Clostridium | 4.88% |
| Coriobacteriaceae | 3.91% |
| vadinCA02 | 3.37% |
| Fusobacterium | 3.36% |
| I025 | 3.31% |
| YS2 | 2.41% |
| PeHg47_OTU27 | 2.21% |
| Parabacteroides_OTU24 | 2.10% |
| Oscillospira_OTU69 | 2.08% |
| Bacteroides_fragilis | 2.00% |
| Boyliae_praeputiale | 1.98% |
| Oscillospira_OTU871 | 1.89% |
| Rikenellaceae | 1.74% |
| Desulfarculaceae_OTU286 | 1.68% |
| Ruminococcaceae_OTU23 | 1.48% |
| Lachnospiraceae_OTU37 | 1.47% |
| Desulfarculaceae_OTU170 | 1.30% |
| Ruminococcus_OTU26 | 1.26% |
| Desulfovibrionaceae | 1.23% |
| Ruminococcaceae_OTU267 | 1.23% |
| Aerococcus_OTU3 | 1.23% |
| Bilophila | 1.17% |
| Enterobacteriaceae_OTU209 | 1.09% |
| Bacteroides_OTU21 | 1.09% |
| Ruminococcaceae_OTU16 | 1.07% |
| Clostridiales_OTU22 | 1.01% |
| Parabacteroides_OTU18 | 0.93% |
| Ruminococcaceae_OTU661 | 0.91% |
| Oscillospira_OTU143 | 0.88% |
| Ruminococcus_AF030449 | 0.80% |
| Lachnospiraceae_OTU10 | 0.77% |
| Oxalobacter_formigene | 0.76% |
| Delftia | 0.71% |
| PeHg47_OTU766 | 0.68% |
| Parabacteroides_OTU266 | 0.64% |
| Propionibacterium_acnes | 0.62% |
| Parabacteroides_OTU375 | 0.45% |
| Parabacteroides_distasonis | 0.44% |

| OTUs | Contribution to CCA 2 |
|---|---|
| Lachnospiraceae_OTU11 | 8.91% |
| Aerococcus_OTU19 | 8.21% |
| Enterobacteriaceae_TOU868 | 7.59% |
| Streptococcus | 6.55% |
| Enterobacteriaceae_OTU29 | 6.50% |
| Parabacteroides_OTU375 | 5.38% |
| Ruminococcaceae_OTU661 | 5.24% |
| Ruminococcaceae_OTU16 | 5.20% |
| Enterobacteriaceae_OTU209 | 3.93% |
| Oscillospira_OTU143 | 3.76% |
| Bacteroides_OTU7 | 3.55% |
| Enterobacteriaceae_OTU731 | 3.46% |
| Peptoniphilus_OTU66 | 3.12% |
| Delftia | 2.52% |
| Flavobacterium | 1.96% |
| Parabacteroides_distasonis | 1.89% |
| Ruminococcus_OTU26 | 1.81% |
| Bacteroides_OTU21 | 1.58% |
| Ruminococcaceae_OTU629 | 1.44% |
| Parabacteroides_OTU266 | 1.24% |
| Ruminococcaceae_OTU589 | 1.18% |
| Oscillospira_OTU855 | 1.10% |
| Aerococcus_OTU1 | 1.08% |
| Ruminococcaceae_OTU267 | 0.98% |
| Lachnospiraceae_OTU10 | 0.93% |
| PeHg47_OTU27 | 0.77% |
| Fusobacterium | 0.70% |
| Oscillospira_OTU69 | 0.68% |
| Ruminococcaceae_OTU23 | 0.65% |
| Campylobacter | 0.61% |
| Clostridiales_OTU22 | 0.59% |
| PeHg47_OTU686 | 0.57% |
| Coriobacteriaceae | 0.56% |
| Synergistaceae | 0.55% |
| PeHg47_OTU766 | 0.51% |
| Propionibacterium_acnes | 0.48% |
| Ruminococcaceae_OTU365 | 0.38% |
| Lonepinella koalarum | 0.37% |
| Propionibacteriaceae_OTU4 | 0.37% |
| Boyliae_praeputiale | 0.31% |
| Ruminococcus_OTU449 | 0.28% |
| Lachnospiraceae_OTU14 | 0.27% |
| Bilophila | 0.27% |
| Phascolarctobacterium | 0.24% |

| | |
|---|---|
| Enterobacteriaceae_OTU731 | 0.37% |
| Lachnospiraceae_OTU726 | 0.36% |
| Lachnospiraceae_OTU11 | 0.33% |
| Streptococcus | 0.29% |
| Propionibacteriaceae_OTU4 | 0.28% |
| Clostridiales_OTU32 | 0.28% |
| Enterobacteriaceae_OTU868 | 0.28% |
| Flavobacterium | 0.23% |
| Ca_Rhodoluna | 0.19% |
| Bacteroides_OTU7 | 0.18% |
| Oscillospira_OTU855 | 0.16% |
| Aerococcus_OTU19 | 0.15% |
| PeHg47_JN680686 | 0.14% |
| Ruminococcaceae_OTU629 | 0.09% |
| Phascolarctobacterium | 0.08% |
| Lachnospiraceae_OTU14 | 0.08% |
| Ruminococcaceae_OTU589 | 0.05% |
| Cytophagaceae | 0.03% |
| Agrobacterium | 0.02% |
| Ruminococcaceae_OTU365 | 0.01% |
| Lonepinella koalarum | 0.01% |
| Enterobacteriaceae_OTU29 | 0.00% |
| Synergistaceae | 0.00% |

| | |
|---|---|
| Ca_Rhodoluna | 0.24% |
| Desulfarculaceae_OTU286 | 0.22% |
| Oscillospira_OTU871 | 0.20% |
| Desulfovibrionaceae | 0.20% |
| Bacteroides_fragilis | 0.14% |
| Lachnospiraceae_OTU726 | 0.14% |
| vadinCA02 | 0.13% |
| Desulfarculaceae_OTU170 | 0.12% |
| Rikenellaceae | 0.08% |
| Aerococcus_OTU3 | 0.08% |
| Lachnospiraceae_OTU37 | 0.06% |
| Parabacteroides_OTU18 | 0.05% |
| Cytophagaceae | 0.02% |
| YS2 | 0.01% |
| Propionibacteriaceae_OTU0 | 0.01% |
| I025 | 0.01% |
| Parabacteroides_OTU24 | 0.01% |
| Clostridium | 0.00% |
| Agrobacterium | 0.00% |
| Clostridiales_OTU32 | 0.00% |
| Facklamia | 0.00% |
| Oxalobacter_formigene | 0.00% |
| Aerococcaceae_OTU31 | 0.00% |

Table S3: Percentage contribution of core OTUs to canonical correspondence analysis of penile microbiome with age, disease and infection-2 as variables (M14).

| OTUs | Contribution to CCA 1 |
|---|---|
| Porphyromonas | 23.11% |
| Peptoniphilus_OTU66 | 14.19% |
| Rs-045 | 8.37% |
| Peptoniphilus_OTU50 | 8.01% |
| Bilophila | 5.76% |
| Campylobacter | 5.58% |
| Aerococcus_OTU19 | 4.81% |
| Aerococcus_OTU208 | 3.59% |
| Synergistaceae | 2.76% |
| Enterobacteriaceae_OTU29 | 2.61% |
| Facklamia | 2.38% |
| Bacteroides_OTU7 | 2.37% |
| Coriobacteriaceae | 2.16% |
| Phascolarctobacterium | 2.03% |
| Aerococcus_OTU1 | 2.01% |
| Aerococcaceae_OTU31 | 1.70% |
| Desulfarculaceae_OTU170 | 1.36% |
| Oscillospira_OTU871 | 1.27% |
| Lachnospiraceae_OTU10 | 1.23% |
| Trueperella | 1.15% |
| Clostridium | 0.87% |
| Aerococcus_OTU3 | 0.61% |
| Flavobacterium | 0.55% |
| Propionibacteriaceae_OTU0 | 0.55% |
| Propionibacterium_acnes | 0.37% |
| Aerococcus_OTU67 | 0.19% |
| Corynebacterium_OTU368 | 0.17% |
| Propionibacteriaceae_OTU4 | 0.12% |
| Corynebacterium_OTU325 | 0.03% |
| Propionibacteriaceae_OTU64 | 0.02% |
| Corynebacterium_OTU390 | 0.02% |
| Aerococcus_OTU207 | 0.00% |
| Boyliae_praeputiale | 0.00% |

| OTUs | Contribution to CCA 2 |
|---|---|
| Aerococcus_OTU3 | 12.80% |
| Trueperella | 11.78% |
| Propionibacteriaceae_OTU64 | 9.73% |
| Flavobacterium | 8.46% |
| Corynebacterium_OTU390 | 8.09% |
| Aerococcus_OTU207 | 7.55% |
| Aerococcus_OTU19 | 5.59% |
| Corynebacterium_OTU325 | 5.26% |
| Aerococcus_OTU208 | 4.83% |
| Synergistaceae | 4.14% |
| Oscillospira_OTU871 | 3.93% |
| Propionibacterium_acnes | 3.56% |
| Bilophila | 2.98% |
| Desulfarculaceae_OTU170 | 2.87% |
| Enterobacteriaceae_OTU29 | 1.63% |
| Lachnospiraceae_OTU10 | 1.56% |
| Propionibacteriaceae_OTU4 | 1.17% |
| Peptoniphilus_OTU50 | 0.74% |
| Boyliae_praeputiale | 0.59% |
| Phascolarctobacterium | 0.51% |
| Bacteroides_OTU7 | 0.47% |
| Corynebacterium_OTU368 | 0.42% |
| Aerococcus_OTU67 | 0.35% |
| Facklamia | 0.32% |
| Campylobacter | 0.29% |
| Aerococcus_OTU1 | 0.15% |
| Propionibacteriaceae_OTU0 | 0.12% |
| Clostridium | 0.07% |
| Coriobacteriaceae | 0.02% |
| Aerococcaceae_OTU31 | 0.01% |
| Rs-045 | 0.01% |
| Peptoniphilus_OTU66 | 0.00% |
| Porphyromonas | 0.00% |

**Table S4: BLAST results for non-*C.pecorum* Chlamydiae identified in the koala urogenital and ocular microbiomes**

| ID | Identity | Coverage | BLAST ID | Accesion number | Source | Family |
|---|---|---|---|---|---|---|
| HF933203 | 98% | 100% | Candidatus *Rhabdochlamydia porcellionis* partial 16S rRNA gene, strain 15C | HF933203 | Isopod | *Rhabdochlamydiaceae* |
| OTU329 | 93% | 99% | Uncultured bacterium partial 16S rRNA gene, isolate Mineral.top.2.1.6_5970 | LN541326 | ground water sand filter | *Rhabdochlamydiaceae* |
| OTU73 | 95% | 100% | Uncultured bacterium clone JK420 16S ribosomal RNA gene | DQ368262 | Black Sea sediment | *Rhabdochlamydiaceae* |
| OTU95 | 90% | 100% | Uncultured bacterium clone M37 16S ribosomal RNA gene | KR0777755 | Marine environment | unknown |
| HG726047 | 99% | 10% | Candidatus *Rubidus massiliensis* partial 16S rRNA gene | HG726047 | ameoba | *Chlamydiaceae* |
| OTU510 | 93% | 100% | Uncultured *Chlamydiae* bacterium clone Upland_75_6831 | JF987533 | Soil | unknown |
| OTU508 | 95% | 100% | Uncultured *Parachlamydia* sp., clone IXZSW1B04HZRBF | LN831108 | Soil | *Parachlamydiaceae* |
| OTU2879 | 94% | 100% | Uncultured bacterium, isolate Mineral.top.1.6.1.4_428898 | LN540733 | ground water sand filter | *Parachlamydiaceae* |
| OTU509 | 95% | 100% | *Parachlamydia* sp. C2, isolate C2 | HG726045 | Amoebal culture from soil | *Parachlamydiaceae* |
| OTU537 | 95% | 100% | Uncultured *Chlamydia* sp., clone IXZSW1B04I32KN | LN831109 | Soil | unknown |
| OTU3241 | 97% | 100% | Uncultured bacterium clone W-Pla-28 | JX279901 | Environmental sample | unknown |

**Table S5: Metadata and alpha diversity metrics for koala urogenital and ocular samples.**

| ID | Site | Sex | Age group | Season | Reproductive status | Disease status | Infection status 2 | Infection status 3 | Chao1 | PD | Shannon | Simpson | ACE |
|---|---|---|---|---|---|---|---|---|---|---|---|---|---|
| KM100 | UGT | Female | Adult | NBS | no-young | RD | positive | High | 179 | 10.3 | 1.88 | 0.64 | 208 |
| KM102 | Penile | Male | Adult | BS | N/A | NAD | positive | Low | 454 | 10.2 | 2.94 | 0.82 | 329 |
| KM104 | Penile | Male | Senior | BS | N/A | NAD | negative | Negative | 70 | 7.5 | 3.21 | 0.86 | 94 |
| KM105 | UGT | Female | Senior | NBS | pouch | NAD | negative | Negative | 121 | 10.6 | 2.65 | 0.63 | 117 |
| KM107 | UGT | Female | SAWJ | NBS | pouch | NAD | negative | Negative | 104 | 12.8 | 4.01 | 0.89 | 121 |
| KM109 | UGT | Female | Senior | NBS | no-young | NAD | positive | Low | 183 | 16.2 | 3.72 | 0.81 | 200 |
| KM112 | UGT | Female | Senior | BS | no-young | RD | negative | Negative | 169 | 11.5 | 3.30 | 0.78 | 135 |
| KM113 | UGT | Female | Senior | NBS | no-young | RD | positive | Low | 198 | 15.7 | 2.62 | 0.64 | 197 |
| KM115 | UGT | Female | Senior | BS | no-young | NAD | positive | Low | 148 | 11.7 | 3.11 | 0.80 | 119 |
| KM118 | UGT | Female | Adult | NBS | no-young | RD | positive | Low | 187 | 16.2 | 2.83 | 0.76 | 172 |
| KM119 | Penile | Male | Senior | BS | N/A | NAD | negative | Negative | 58 | 6.4 | 2.80 | 0.82 | 78 |
| KM122 | UGT | Female | SAWJ | NBS | pouch | NAD | negative | Negative | 102 | 11.2 | 3.71 | 0.85 | 111 |
| KM124 | UGT | Female | Senior | BS | no-young | RD | negative | Negative | 88 | 7.4 | 2.37 | 0.68 | 98 |
| KM125 | UGT | Female | Adult | NBS | no-young | NAD | positive | High | 142 | 13.8 | 1.95 | 0.59 | 183 |
| KM127 | Penile | Male | Adult | NBS | N/A | RD | negative | Negative | 68 | 10.1 | 2.67 | 0.69 | 72 |
| KM129 | Penile | Male | Adult | NBS | N/A | RD | negative | Negative | 75 | 5.0 | 2.52 | 0.78 | 131 |
| KM130 | UGT | Female | Adult | NBS | pap | NAD | negative | Negative | 181 | 10.2 | 3.96 | 0.89 | 153 |
| KM133 | UGT | Female | Adult | NBS | no-young | RD | negative | Negative | 178 | 12.8 | 3.05 | 0.79 | 205 |
| KM134 | Penile | Male | Adult | NBS | N/A | NAD | negative | Negative | 94 | 8.3 | 3.06 | 0.85 | 123 |
| KM136 | Penile | Male | Adult | NBS | N/A | NAD | positive | Low | 207 | 10.7 | 2.70 | 0.79 | 266 |
| KM138 | Penile | Male | Senior | NBS | N/A | RD | positive | High | 250 | 11.0 | 3.22 | 0.83 | 231 |
| KM140 | UGT | Female | Adult | BS | pouch | NAD | negative | Negative | 219 | 13.1 | 1.44 | 0.39 | 216 |
| KM141 | UGT | Female | Adult | NBS | pap | NAD | positive | Low | 279 | 21.2 | 4.12 | 0.85 | 271 |
| KM142 | Ocular | Female | Adult | NBS | pouch | NAD | negative | negative | 966 | 55.6 | 7.79 | 0.99 | 1010 |
| KM143 | Penile | Male | Adult | NBS | N/A | NAD | negative | Negative | 237 | 6.3 | 1.39 | 0.48 | 372 |
| KM147 | Penile | Male | SA | NBS | N/A | RD | negative | Negative | 161 | 14.1 | 3.20 | 0.84 | 219 |
| KM148 | UGT | Female | SA | BS | no-young | NAD | negative | Negative | 262 | 18.6 | 2.77 | 0.65 | 331 |
| KM149 | Penile | Male | Adult | BS | N/A | NAD | negative | Negative | 103 | 8.6 | 2.78 | 0.82 | 111 |
| KM151 | UGT | Female | Senior | NBS | no-young | RD | positive | High | 100 | 9.0 | 1.52 | 0.39 | 111 |
| KM155 | UGT | Female | Adult | BS | pouch | NAD | negative | Negative | 141 | 12.8 | 3.93 | 0.87 | 131 |
| KM157 | UGT | Female | Adult | NBS | no-young | NAD | positive | High | 53 | 7.0 | 1.72 | 0.56 | 70 |
| KM159 | Penile | Male | Adult | BS | N/A | NAD | negative | Negative | 202 | 17.6 | 4.09 | 0.83 | 215 |
| KM161 | UGT | Female | Adult | NBS | pap | NAD | negative | Negative | 130 | 11.1 | 1.33 | 0.36 | 137 |
| KM163 | UGT | Female | Adult | NBS | no-young | RD | negative | Low | 270 | 16.9 | 3.84 | 0.86 | 301 |
| KM164 | UGT | Female | Senior | NBS | no-young | RD | positive | Low | 125 | 13.1 | 1.88 | 0.47 | 150 |
| KM165 | UGT | Female | SAWJ | NBS | pouch | NAD | negative | Negative | 288 | 22.6 | 3.20 | 0.77 | 283 |
| KM167 | UGT | Female | Adult | NBS | back | NAD | negative | Negative | 154 | 13.4 | 0.78 | 0.16 | 193 |
| KM169 | UGT | Female | SAWJ | NBS | pouch | NAD | negative | Negative | 199 | 22.1 | 4.04 | 0.85 | 204 |
| KM173 | Penile | Male | Adult | NBS | N/A | RD | positive | Low | 160 | 9.3 | 2.84 | 0.82 | 241 |
| KM174 | UGT | Female | Adult | NBS | pouch | NAD | negative | Negative | 172 | 13.9 | 3.23 | 0.76 | 192 |
| KM176 | Penile | Male | Adult | NBS | N/A | NAD | negative | Negative | 178 | 16.0 | 2.50 | 0.74 | 194 |

| | | | | | | | | | | | | | |
|---|---|---|---|---|---|---|---|---|---|---|---|---|---|
| KM178 | Penile | Male | Senior | BS | N/A | NAD | negative | Negative | 99 | 6.5 | 2.72 | 0.81 | 87 |
| KM179 | UGT | Female | Senior | BS | no-young | RD | positive | Low | 153 | 14.4 | 3.20 | 0.74 | 176 |
| KM180 | UGT | Female | SA | BS | no-young | RD | positive | Low | 142 | 12.2 | 4.75 | 0.94 | 127 |
| KM181 | UGT | Female | Adult | NBS | no-young | NAD | positive | Low | 180 | 10.1 | 3.13 | 0.72 | 186 |
| KM183 | Penile | Male | SA | NBS | N/A | NAD | negative | Negative | 101 | 6.3 | 2.27 | 0.72 | 152 |
| KM185 | UGT | Female | Adult | NBS | no-young | NAD | negative | Negative | 87 | 8.0 | 1.63 | 0.44 | 91 |
| KM187 | UGT | Female | Senior | BS | pouch | NAD | negative | Negative | 198 | 15.6 | 3.80 | 0.86 | 204 |
| KM189 | Penile | Male | SA | BS | N/A | NAD | negative | Negative | 69 | 6.9 | 2.04 | 0.70 | 84 |
| KM191 | UGT | Female | Senior | BS | pouch | NAD | negative | Negative | 151 | 13.7 | 2.82 | 0.71 | 167 |
| KM195 | Penile | Male | SA | NBS | N/A | RD | positive | Low | 92 | 9.0 | 2.46 | 0.71 | 114 |
| KM196 | UGT | Female | Adult | NBS | pouch | NAD | negative | Negative | 124 | 10.7 | 3.11 | 0.84 | 126 |
| KM20 | Ocular | Female | SAWJ | NBS | pouch | NAD | negative | negative | 125 | 12.4 | 3.40 | 0.81 | 122 |
| KM200 | Penile | Male | Adult | NBS | N/A | NAD | negative | Negative | 74 | 6.8 | 3.32 | 0.88 | 107 |
| KM203 | Penile | Male | Senior | NBS | N/A | NAD | negative | Negative | 247 | 11.2 | 1.86 | 0.64 | 250 |
| KM205 | UGT | Female | Adult | NBS | no-young | RD | positive | High | 93 | 9.5 | 0.84 | 0.21 | 103 |
| KM207 | UGT | Female | Adult | NBS | no-young | RD | positive | Low | 184 | 13.8 | 3.81 | 0.87 | 171 |
| KM209 | UGT | Female | Adult | BS | pap | NAD | negative | Negative | 231 | 21.2 | 2.69 | 0.70 | 264 |
| KM211 | UGT | Female | SA | BS | no-young | NAD | negative | Negative | 117 | 12.0 | 1.72 | 0.43 | 135 |
| KM213 | Penile | Male | SA | NBS | N/A | NAD | negative | Negative | 52 | 7.2 | 1.57 | 0.62 | 59 |
| KM215 | Penile | Male | SA | BS | N/A | NAD | negative | Negative | 199 | 14.8 | 4.34 | 0.91 | 220 |
| KM216 | Ocular | Male | SA | BS | N/A | NAD | negative | negative | 280 | 25.9 | 5.89 | 0.96 | 282 |
| KM217 | UGT | Female | SAWJ | BS | back | NAD | negative | Negative | 190 | 14.1 | 4.08 | 0.87 | 216 |
| KM218 | Penile | Male | Senior | NBS | N/A | RD | positive | Low | 125 | 9.4 | 2.83 | 0.80 | 132 |
| KM219 | UGT | Female | SA | NBS | no-young | NAD | negative | Negative | 93 | 9.4 | 1.82 | 0.44 | 106 |
| KM221 | UGT | Female | Senior | NBS | no-young | RD | positive | Low | 99 | 7.4 | 1.25 | 0.49 | 64 |
| KM223 | UGT | Female | Adult | NBS | pouch | NAD | negative | Negative | 151 | 7.7 | 3.08 | 0.84 | 187 |
| KM225 | UGT | Female | Senior | BS | back | NAD | negative | Negative | 90 | 10.0 | 2.90 | 0.78 | 102 |
| KM229 | UGT | Female | Senior | NBS | pap | NAD | negative | Negative | 173 | 14.9 | 2.32 | 0.67 | 210 |
| KM232 | Ocular | Female | Senior | NBS | pouch | NAD | negative | negative | 223 | 14.7 | 2.91 | 0.76 | 178 |
| KM239 | Penile | Male | Adult | NBS | N/A | NAD | positive | Low | 147 | 13.1 | 3.34 | 0.86 | 158 |
| KM241 | UGT | Female | Adult | BS | no-young | RD | negative | Negative | 95 | 9.8 | 2.62 | 0.71 | 82 |
| KM242 | Penile | Male | SA | BS | N/A | NAD | negative | Negative | 83 | 5.3 | 2.27 | 0.68 | 73 |
| KM243 | Penile | Male | Senior | NBS | N/A | NAD | negative | Negative | 67 | 6.3 | 2.38 | 0.70 | 72 |
| KM244 | UGT | Female | SA | BS | no-young | RD | negative | Negative | 183 | 11.9 | 4.09 | 0.87 | 167 |
| KM245 | UGT | Female | Adult | BS | no-young | RD | positive | High | 99 | 9.7 | 2.68 | 0.74 | 87 |
| KM246 | UGT | Female | Adult | NBS | back | NAD | negative | Negative | 107 | 10.9 | 3.95 | 0.89 | 100 |
| KM247 | Penile | Male | Adult | NBS | N/A | NAD | negative | Negative | 161 | 13.9 | 3.02 | 0.85 | 185 |
| KM248 | UGT | Female | Adult | BS | back | NAD | negative | Negative | 96 | 8.6 | 3.06 | 0.83 | 95 |
| KM250 | Penile | Male | Adult | NBS | N/A | NAD | negative | Negative | 56 | 7.5 | 2.49 | 0.69 | 56 |
| KM251 | UGT | Female | Senior | NBS | no-young | NAD | positive | Low | 55 | 6.8 | 2.77 | 0.77 | 76 |
| KM252 | Penile | Male | Adult | NBS | N/A | RD | negative | Negative | 133 | 12.8 | 3.81 | 0.88 | 121 |
| KM253 | Ocular | Male | Adult | NBS | N/A | NAD | negative | negative | 353 | 26.3 | 5.75 | 0.96 | 365 |
| KM254 | UGT | Female | SAWJ | NBS | pouch | NAD | negative | Negative | 244 | 14.3 | 4.41 | 0.91 | 230 |
| KM255 | Ocular | Female | SAWJ | NBS | pouch | NAD | negative | negative | 387 | 32.3 | 6.05 | 0.96 | 395 |
| KM258 | UGT | Female | Senior | NBS | no-young | RD | positive | Low | 80 | 10.6 | 3.62 | 0.80 | 85 |

| | | | | | | | | | | | | | |
|---|---|---|---|---|---|---|---|---|---|---|---|---|---|
| KM259 | UGT | Female | Senior | NBS | pouch | NAD | negative | Negative | 118 | 10.4 | 1.57 | 0.39 | 146 |
| KM26 | UGT | Female | Senior | NBS | back | NAD | negative | Negative | 79 | 10.2 | 3.12 | 0.79 | 100 |
| KM261 | Penile | Male | Adult | BS | N/A | RD | negative | Negative | 32 | 5.9 | 2.21 | 0.67 | 31 |
| KM262 | UGT | Female | SAWJ | BS | pap | NAD | negative | Negative | 153 | 13.0 | 4.12 | 0.87 | 154 |
| KM264 | UGT | Female | Adult | BS | no-young | RD | negative | Negative | 164 | 15.7 | 3.49 | 0.83 | 174 |
| KM265 | UGT | Female | SA | BS | no-young | NAD | negative | Negative | 201 | 16.7 | 3.66 | 0.85 | 210 |
| KM266 | UGT | Female | Adult | BS | no-young | NAD | positive | Low | 213 | 19.1 | 2.90 | 0.62 | 210 |
| KM268 | Penile | Male | Senior | BS | N/A | NAD | negative | Negative | 207 | 7.6 | 2.77 | 0.82 | 88 |
| KM269 | UGT | Female | SA | NBS | no-young | NAD | negative | Negative | 110 | 7.7 | 2.25 | 0.64 | 115 |
| KM27 | UGT | Female | Adult | NBS | pap | NAD | negative | Negative | 44 | 5.9 | 1.51 | 0.52 | 36 |
| KM273 | Penile | Male | Adult | NBS | N/A | NAD | negative | Negative | 66 | 5.8 | 2.75 | 0.79 | 89 |
| KM277 | UGT | Female | Adult | NBS | no-young | NAD | negative | Negative | 113 | 12.0 | 4.08 | 0.90 | 103 |
| KM278 | UGT | Female | Adult | NBS | no-young | RD | negative | Negative | 123 | 12.7 | 4.01 | 0.85 | 121 |
| KM279 | Penile | Male | Adult | NBS | N/A | NAD | negative | Negative | 88 | 8.7 | 2.33 | 0.70 | 120 |
| KM280 | Ocular | Male | Adult | NBS | N/A | EI | negative | negative | 300 | 24.5 | 2.23 | 0.62 | 346 |
| KM281 | Penile | Male | Adult | NBS | N/A | NAD | negative | Negative | 132 | 10.6 | 2.81 | 0.78 | 143 |
| KM283 | Penile | Male | Adult | BS | N/A | NAD | negative | Negative | 110 | 8.5 | 2.23 | 0.69 | 118 |
| KM285 | Penile | Male | SA | NBS | N/A | NAD | negative | Negative | 45 | 6.9 | 2.00 | 0.65 | 41 |
| KM288 | Ocular | Male | SA | NBS | N/A | NAD | negative | negative | 495 | 39.0 | 5.93 | 0.95 | 495 |
| KM291 | UGT | Female | Adult | BS | no-young | RD | negative | Negative | 165 | 13.0 | 4.03 | 0.90 | 181 |
| KM292 | UGT | Female | SAWJ | NBS | pouch | NAD | negative | Negative | 160 | 14.3 | 3.74 | 0.84 | 162 |
| KM294 | Penile | Male | SA | NBS | N/A | NAD | negative | Negative | 139 | 10.4 | 0.82 | 0.17 | 157 |
| KM296 | UGT | Female | SA | BS | no-young | NAD | negative | Negative | 77 | 7.6 | 3.23 | 0.87 | 67 |
| KM298 | Penile | Male | SA | NBS | N/A | NAD | positive | Low | 27 | 3.7 | 2.71 | 0.80 | 41 |
| KM30 | UGT | Female | SA | NBS | no-young | RD | positive | High | 49 | 6.1 | 1.02 | 0.28 | 69 |
| KM300 | Penile | Male | Senior | BS | N/A | NAD | negative | Negative | 120 | 9.4 | 1.88 | 0.42 | 129 |
| KM301 | UGT | Female | SAWJ | NBS | pap | NAD | negative | Negative | 332 | 15.2 | 3.05 | 0.80 | 284 |
| KM305 | UGT | Female | SAWJ | BS | pouch | NAD | negative | Negative | 251 | 16.4 | 4.60 | 0.93 | 267 |
| KM307 | Penile | Male | Adult | NBS | N/A | RD | negative | Negative | 158 | 16.5 | 2.83 | 0.73 | 173 |
| KM308 | UGT | Female | Senior | NBS | no-young | RD | positive | Low | 87 | 9.9 | 4.19 | 0.92 | 79 |
| KM309 | Ocular | Female | Senior | NBS | no_young | NAD | negative | negative | 479 | 39.7 | 7.29 | 0.99 | 495 |
| KM310 | UGT | Female | SAWJ | NBS | pouch | NAD | negative | Negative | 177 | 13.7 | 4.25 | 0.92 | 169 |
| KM312 | Penile | Male | SA | NBS | N/A | RD | positive | Low | 204 | 7.7 | 2.70 | 0.80 | 237 |
| KM313 | UGT | Female | Adult | BS | back | NAD | negative | Negative | 64 | 5.9 | 1.96 | 0.65 | 74 |
| KM315 | Penile | Male | Senior | NBS | N/A | NAD | negative | Negative | 82 | 9.2 | 2.93 | 0.81 | 81 |
| KM317 | UGT | Female | Adult | BS | Pregnant | NAD | negative | Negative | 121 | 8.1 | 1.76 | 0.60 | 93 |
| KM318 | UGT | Female | Adult | NBS | back | NAD | negative | Negative | 171 | 12.4 | 2.96 | 0.80 | 145 |
| KM32 | Penile | Male | SA | BS | N/A | NAD | negative | Negative | 79 | 6.2 | 2.92 | 0.85 | 85 |
| KM320 | UGT | Female | SA | NBS | no-young | NAD | negative | Negative | 179 | 18.1 | 2.81 | 0.69 | 212 |
| KM322 | UGT | Female | SA | NBS | no-young | NAD | negative | Negative | 194 | 10.7 | 3.41 | 0.83 | 154 |
| KM324 | UGT | Female | Senior | BS | no-young | NAD | negative | Negative | 157 | 13.1 | 4.12 | 0.90 | 172 |
| KM326 | Penile | Male | Adult | NBS | N/A | NAD | negative | Negative | 89 | 7.6 | 2.85 | 0.80 | 114 |
| KM328 | UGT | Female | SA | BS | no-young | RD | positive | High | 56 | 6.7 | 2.30 | 0.75 | 68 |
| KM33 | Penile | Male | SA | NBS | N/A | NAD | positive | High | 89 | 6.2 | 2.20 | 0.69 | 81 |
| KM331 | UGT | Female | Adult | NBS | no-young | RD | negative | Negative | 199 | 12.6 | 3.12 | 0.83 | 175 |

| | | | | | | | | | | | | | |
|---|---|---|---|---|---|---|---|---|---|---|---|---|---|
| KM332 | Penile | Male | SA | NBS | N/A | NAD | negative | Negative | 55 | 6.5 | 2.77 | 0.80 | 68 |
| KM333 | UGT | Female | Adult | BS | no-young | RD | negative | Negative | 173 | 11.2 | 2.44 | 0.69 | 188 |
| KM334 | UGT | Female | SA | BS | no-young | NAD | negative | Negative | 96 | 11.7 | 2.61 | 0.65 | 108 |
| KM336 | Penile | Male | SA | BS | N/A | NAD | negative | Negative | 94 | 8.7 | 1.82 | 0.53 | 95 |
| KM338 | Penile | Male | Adult | NBS | N/A | NAD | negative | Negative | 83 | 6.4 | 2.79 | 0.83 | 88 |
| KM34 | Penile | Male | SA | NBS | N/A | NAD | negative | Negative | 21 | 3.9 | 2.21 | 0.69 | 23 |
| KM340 | Penile | Male | SA | BS | N/A | NAD | negative | Negative | 93 | 7.5 | 1.77 | 0.53 | 191 |
| KM341 | Penile | Male | Adult | BS | N/A | NAD | negative | Negative | 100 | 6.3 | 2.56 | 0.77 | 103 |
| KM343 | UGT | Female | SA | BS | no-young | NAD | negative | Negative | 88 | 6.4 | 0.14 | 0.03 | 96 |
| KM344 | UGT | Female | Adult | BS | no-young | NAD | positive | Low | 93 | 10.2 | 3.15 | 0.76 | 100 |
| KM345 | UGT | Female | SA | NBS | no-young | NAD | negative | Negative | 269 | 17.3 | 2.31 | 0.63 | 307 |
| KM347 | Penile | Male | SA | NBS | N/A | NAD | negative | Negative | 152 | 7.0 | 2.92 | 0.84 | 154 |
| KM349 | Penile | Male | SA | NBS | N/A | NAD | negative | Negative | 175 | 9.1 | 2.09 | 0.63 | 251 |
| KM35 | UGT | Female | Senior | BS | no-young | NAD | positive | Low | 29 | 3.8 | 2.47 | 0.77 | 35 |
| KM351 | UGT | Female | SA | NBS | no-young | NAD | negative | Negative | 244 | 23.2 | 4.98 | 0.94 | 250 |
| KM355 | UGT | Female | Adult | BS | no-young | RD | negative | Negative | 96 | 7.9 | 1.67 | 0.44 | 98 |
| KM356 | Penile | Male | Adult | BS | N/A | NAD | negative | Negative | 77 | 6.3 | 2.85 | 0.81 | 83 |
| KM357 | Ocular | Male | Adult | BS | N/A | NAD | negative | negative | 350 | 30.2 | 2.66 | 0.55 | 330 |
| KM358 | Penile | Male | Senior | NBS | N/A | NAD | negative | Negative | 89 | 6.9 | 3.06 | 0.85 | 101 |
| KM359 | Ocular | Male | Senior | NBS | N/A | EI | negative | negative | 377 | 21.0 | 4.65 | 0.90 | 329 |
| KM360 | Penile | Male | Adult | BS | N/A | RD | negative | Negative | 99 | 8.3 | 0.44 | 0.08 | 99 |
| KM361 | Penile | Male | Senior | NBS | N/A | RD | negative | Negative | 146 | 11.2 | 2.34 | 0.72 | 135 |
| KM362 | Penile | Male | Adult | NBS | N/A | NAD | positive | Low | 86 | 6.6 | 3.11 | 0.85 | 110 |
| KM365 | UGT | Female | Adult | NBS | pap | NAD | negative | Negative | 190 | 13.2 | 3.47 | 0.82 | 215 |
| KM368 | UGT | Female | Adult | NBS | back | NAD | negative | Negative | 96 | 8.0 | 3.15 | 0.86 | 109 |
| KM371 | UGT | Female | Adult | NBS | back | NAD | negative | Negative | 223 | 12.2 | 3.50 | 0.87 | 160 |
| KM373 | Penile | Male | SA | NBS | N/A | RD | positive | Low | 94 | 8.0 | 1.62 | 0.57 | 98 |
| KM374 | Penile | Male | Adult | NBS | N/A | NAD | negative | Negative | 111 | 7.4 | 2.25 | 0.71 | 129 |
| KM375 | UGT | Female | Adult | NBS | no-young | RD | negative | Negative | 54 | 6.9 | 1.57 | 0.59 | 78 |
| KM376 | Penile | Male | Adult | BS | N/A | NAD | positive | Low | 63 | 7.0 | 3.02 | 0.83 | 84 |
| KM38 | UGT | Female | Adult | NBS | pap | NAD | negative | Negative | 183 | 12.3 | 2.83 | 0.70 | 168 |
| KM382 | Penile | Male | Adult | BS | N/A | NAD | positive | Low | 284 | 15.4 | 3.99 | 0.89 | 282 |
| KM383 | Ocular | Male | Adult | BS | N/A | NAD | negative | negative | 300 | 22.7 | 2.44 | 0.57 | 336 |
| KM384 | UGT | Female | Senior | BS | pouch | NAD | negative | Negative | 89 | 9.1 | 2.74 | 0.78 | 96 |
| KM389 | UGT | Female | Adult | BS | pouch | NAD | negative | Negative | 149 | 9.8 | 3.10 | 0.82 | 129 |
| KM390 | Ocular | Female | Adult | BS | pouch | EI | negative | negative | 375 | 25.4 | 2.92 | 0.76 | 398 |
| KM391 | UGT | Female | SA | BS | no-young | RD | positive | High | 64 | 8.8 | 3.24 | 0.86 | 90 |
| KM393 | Penile | Male | SA | BS | N/A | NAD | negative | Negative | 50 | 5.4 | 2.95 | 0.84 | 63 |
| KM395 | UGT | Female | Adult | BS | back | NAD | negative | Negative | 272 | 15.5 | 4.60 | 0.93 | 235 |
| KM397 | Penile | Male | SA | BS | N/A | NAD | negative | Negative | 106 | 7.4 | 1.96 | 0.62 | 95 |
| KM398 | UGT | Female | SAWJ | BS | Pregnant | NAD | negative | Negative | 90 | 10.2 | 2.98 | 0.76 | 78 |
| KM399 | UGT | Female | SA | BS | no-young | NAD | negative | Negative | 93 | 9.3 | 3.14 | 0.81 | 90 |
| KM400 | UGT | Female | SAWJ | BS | back | NAD | negative | Negative | 53 | 6.5 | 2.25 | 0.71 | 50 |
| KM403 | UGT | Female | Senior | BS | no-young | NAD | negative | Negative | 299 | 19.0 | 3.27 | 0.78 | 319 |
| KM405 | Penile | Male | Adult | BS | N/A | NAD | negative | Negative | 84 | 9.6 | 1.90 | 0.58 | 95 |

| | | | | | | | | | | | | | |
|---|---|---|---|---|---|---|---|---|---|---|---|---|---|
| KM406 | Ocular | Male | Adult | BS | N/A | NAD | negative | negative | 417 | 31.9 | 6.16 | 0.96 | 405 |
| KM407 | Penile | Male | Adult | BS | N/A | NAD | negative | Negative | 69 | 9.4 | 2.83 | 0.82 | 83 |
| KM409 | Penile | Male | Adult | BS | N/A | NAD | negative | Negative | 88 | 7.0 | 2.82 | 0.79 | 89 |
| KM411 | Penile | Male | Adult | BS | N/A | RD | negative | Negative | 33 | 4.9 | 2.80 | 0.80 | 35 |
| KM414 | Penile | Male | Senior | BS | N/A | NAD | negative | Negative | 85 | 5.9 | 3.12 | 0.85 | 78 |
| KM415 | Penile | Male | Adult | BS | N/A | NAD | negative | Negative | 117 | 6.8 | 2.58 | 0.79 | 154 |
| KM42 | Penile | Male | SA | NBS | N/A | NAD | negative | Negative | 13 | 2.1 | 2.19 | 0.71 | 18 |
| KM420 | UGT | Female | SAWJ | BS | Pregnant | RD | positive | High | 44 | 6.7 | 0.61 | 0.16 | 49 |
| KM421 | Ocular | Female | SAWJ | BS | pregnant | NAD | negative | negative | 114 | 10.4 | 1.57 | 0.32 | 112 |
| KM426 | Penile | Male | Adult | BS | N/A | RD | positive | Low | 93 | 8.2 | 3.12 | 0.83 | 90 |
| KM427 | Penile | Male | Adult | BS | N/A | NAD | negative | Negative | 139 | 11.1 | 3.34 | 0.87 | 162 |
| KM428 | Ocular | Male | Adult | BS | N/A | NAD | negative | negative | 399 | 31.7 | 6.15 | 0.97 | 405 |
| KM429 | UGT | Female | SA | NBS | no-young | NAD | negative | Negative | 232 | 16.0 | 3.86 | 0.87 | 240 |
| KM43 | UGT | Female | Senior | BS | pouch | NAD | negative | Negative | 169 | 9.8 | 2.96 | 0.81 | 142 |
| KM431 | UGT | Female | Senior | NBS | back | NAD | negative | Negative | 154 | 12.0 | 2.96 | 0.81 | 163 |
| KM433 | Penile | Male | SA | NBS | N/A | NAD | negative | Negative | 115 | 6.9 | 1.96 | 0.62 | 128 |
| KM438 | UGT | Female | Adult | BS | no-young | RD | negative | Negative | 94 | 9.3 | 4.32 | 0.92 | 97 |
| KM439 | UGT | Female | SA | BS | no-young | NAD | negative | Negative | 157 | 14.5 | 3.72 | 0.87 | 178 |
| KM44 | UGT | Female | Adult | BS | pap | NAD | negative | Negative | 202 | 10.5 | 2.79 | 0.79 | 267 |
| KM448 | UGT | Female | Senior | BS | no-young | RD | positive | High | 160 | 9.7 | 2.36 | 0.71 | 147 |
| KM449 | Penile | Male | Adult | BS | N/A | RD | positive | Low | 205 | 7.9 | 2.64 | 0.75 | 166 |
| KM45 | UGT | Female | Adult | BS | Pregnant | RD | positive | High | 99 | 7.6 | 2.28 | 0.69 | 129 |
| KM450 | UGT | Female | SA | NBS | no-young | NAD | negative | Negative | 62 | 8.0 | 1.82 | 0.45 | 61 |
| KM452 | UGT | Female | SA | NBS | no-young | NAD | negative | Negative | 110 | 12.5 | 2.37 | 0.65 | 117 |
| KM454 | Penile | Male | Adult | BS | N/A | RD | positive | Low | 140 | 8.3 | 3.03 | 0.85 | 327 |
| KM455 | UGT | Female | Adult | BS | no-young | RD | positive | High | 97 | 8.2 | 3.16 | 0.84 | 102 |
| KM456 | UGT | Female | SA | BS | no-young | NAD | negative | Negative | 63 | 7.6 | 2.06 | 0.57 | 74 |
| KM457 | UGT | Female | Adult | BS | no-young | RD | negative | Negative | 111 | 12.1 | 4.20 | 0.90 | 113 |
| KM458 | UGT | Female | Adult | NBS | no-young | RD | positive | Low | 135 | 12.3 | 3.72 | 0.87 | 143 |
| KM459 | Penile | Male | Senior | BS | N/A | NAD | negative | Negative | 44 | 5.4 | 2.25 | 0.70 | 86 |
| KM46 | Penile | Male | Adult | BS | N/A | RD | positive | High | 230 | 16.2 | 3.16 | 0.83 | 202 |
| KM460 | UGT | Female | Adult | NBS | pouch | NAD | negative | Negative | 157 | 14.9 | 4.50 | 0.93 | 160 |
| KM462 | UGT | Female | Adult | BS | back | NAD | negative | Negative | 167 | 10.6 | 2.44 | 0.72 | 193 |
| KM472 | Penile | Male | SA | BS | N/A | NAD | negative | Negative | 150 | 7.8 | 2.10 | 0.70 | 155 |
| KM473 | Penile | Male | SA | BS | N/A | NAD | positive | High | 68 | 9.5 | 3.33 | 0.85 | 68 |
| KM475 | Penile | Male | Senior | NBS | N/A | RD | positive | High | 148 | 7.3 | 2.75 | 0.80 | 123 |
| KM476 | Penile | Male | SA | BS | N/A | NAD | negative | Negative | 169 | 11.9 | 1.36 | 0.41 | 131 |
| KM477 | UGT | Female | Adult | BS | no-young | RD | positive | Low | 238 | 8.4 | 2.02 | 0.57 | 318 |
| KM478 | UGT | Female | Senior | NBS | no-young | NAD | negative | Negative | 152 | 13.9 | 3.69 | 0.87 | 156 |
| KM48 | UGT | Female | Adult | BS | no-young | NAD | negative | Negative | 171 | 9.0 | 0.42 | 0.09 | 122 |
| KM480 | Penile | Male | Senior | BS | N/A | NAD | negative | Negative | 132 | 8.1 | 2.92 | 0.84 | 169 |
| KM483 | Penile | Male | Senior | NBS | N/A | NAD | negative | Negative | 74 | 8.0 | 2.86 | 0.83 | 78 |
| KM484 | Penile | Male | SA | BS | N/A | NAD | negative | Negative | 48 | 6.5 | 1.42 | 0.51 | 58 |
| KM487 | Ocular | Female | SA | BS | no_young | NAD | negative | negative | 148 | 12.7 | 2.64 | 0.70 | 155 |
| KM49 | Penile | Male | Adult | BS | N/A | NAD | negative | Negative | 296 | 16.3 | 3.72 | 0.88 | 324 |

| | | | | | | | | | | | | | |
|---|---|---|---|---|---|---|---|---|---|---|---|---|---|
| KM490 | Penile | Male | Adult | BS | N/A | RD | negative | Negative | 42 | 4.8 | 0.34 | 0.08 | 31 |
| KM492 | Penile | Male | Adult | BS | N/A | NAD | negative | Negative | 72 | 5.7 | 2.72 | 0.81 | 69 |
| KM493 | Penile | Male | SA | BS | N/A | RD | negative | Negative | 227 | 9.9 | 1.79 | 0.54 | 220 |
| KM494 | UGT | Female | SA | BS | no-young | NAD | negative | Negative | 102 | 12.7 | 2.49 | 0.75 | 123 |
| KM495 | Penile | Male | Adult | BS | N/A | NAD | negative | Negative | 86 | 7.8 | 3.11 | 0.86 | 103 |
| KM497 | UGT | Female | SA | BS | no-young | NAD | negative | Negative | 84 | 8.6 | 2.71 | 0.76 | 85 |
| KM498 | Penile | Male | Adult | BS | N/A | NAD | negative | Negative | 38 | 5.0 | 2.98 | 0.84 | 70 |
| KM499 | Penile | Male | Adult | BS | N/A | RD | positive | High | 65 | 6.1 | 1.89 | 0.54 | 126 |
| KM500 | UGT | Female | Adult | BS | no-young | RD | positive | Low | 195 | 12.0 | 3.45 | 0.84 | 168 |
| KM501 | Penile | Male | SA | BS | N/A | NAD | negative | Negative | 100 | 10.5 | 2.03 | 0.55 | 103 |
| KM508 | UGT | Female | SA | BS | no-young | NAD | negative | Negative | 130 | 11.9 | 4.22 | 0.93 | 135 |
| KM510 | Penile | Male | Adult | BS | N/A | NAD | negative | Negative | 96 | 7.6 | 2.13 | 0.72 | 104 |
| KM513 | UGT | Female | Senior | BS | no-young | NAD | negative | Negative | 92 | 10.4 | 4.31 | 0.90 | 98 |
| KM518 | UGT | Female | SAWJ | BS | pouch | NAD | negative | Negative | 296 | 13.8 | 4.52 | 0.91 | 195 |
| KM519 | UGT | Female | Senior | BS | pouch | NAD | negative | Negative | 92 | 8.1 | 1.53 | 0.39 | 77 |
| KM525 | Penile | Male | Adult | BS | N/A | NAD | positive | High | 105 | 7.7 | 1.92 | 0.57 | 125 |
| KM527 | UGT | Female | SA | BS | no-young | NAD | negative | Negative | 74 | 7.1 | 1.87 | 0.57 | 104 |
| KM528 | UGT | Female | Adult | BS | pouch | NAD | negative | Negative | 170 | 13.9 | 3.81 | 0.83 | 165 |
| KM529 | UGT | Female | Senior | BS | no-young | NAD | negative | Negative | 192 | 14.5 | 3.80 | 0.84 | 216 |
| KM530 | UGT | Female | Adult | BS | pouch | NAD | negative | Negative | 75 | 11.1 | 1.88 | 0.51 | 84 |
| KM531 | UGT | Female | Adult | BS | no-young | NAD | negative | Negative | 139 | 11.9 | 2.91 | 0.75 | 163 |
| KM532 | Penile | Male | Adult | BS | N/A | NAD | negative | Negative | 98 | 6.4 | 2.82 | 0.83 | 110 |
| KM534 | Penile | Male | SA | BS | N/A | NAD | negative | Negative | 61 | 8.7 | 2.74 | 0.80 | 66 |
| KM535 | UGT | Female | Adult | BS | pouch | NAD | negative | Negative | 220 | 11.8 | 4.10 | 0.90 | 154 |
| KM536 | UGT | Female | SA | BS | no-young | NAD | negative | Negative | 80 | 10.9 | 2.88 | 0.77 | 85 |
| KM541 | UGT | Female | Adult | BS | Pregnant | RD | negative | Negative | 85 | 5.8 | 2.22 | 0.67 | 83 |
| KM542 | UGT | Female | Adult | BS | no-young | RD | negative | Negative | 131 | 6.6 | 3.54 | 0.88 | 93 |
| KM543 | Penile | Male | Adult | BS | N/A | NAD | negative | Negative | 50 | 6.2 | 1.31 | 0.33 | 58 |
| KM544 | UGT | Female | Senior | BS | no-young | NAD | positive | Low | 88 | 9.9 | 3.59 | 0.84 | 81 |
| KM545 | UGT | Female | Adult | BS | no-young | RD | positive | Low | 110 | 10.7 | 2.70 | 0.61 | 122 |
| KM546 | UGT | Female | Adult | BS | pouch | NAD | negative | Negative | 83 | 9.0 | 2.74 | 0.73 | 85 |
| KM547 | UGT | Female | Adult | BS | pouch | NAD | negative | Negative | 189 | 13.9 | 3.81 | 0.83 | 197 |
| KM548 | Penile | Male | SA | NBS | N/A | NAD | negative | Negative | 77 | 6.4 | 1.77 | 0.51 | 101 |
| KM58 | UGT | Female | Adult | BS | Pregnant | RD | positive | High | 35 | 5.0 | 1.97 | 0.66 | 44 |
| KM59 | Penile | Male | SA | BS | N/A | NAD | negative | Negative | 39 | 4.2 | 2.59 | 0.77 | 67 |
| KM61 | Penile | Male | SA | BS | N/A | NAD | negative | Negative | 142 | 9.1 | 2.94 | 0.84 | 261 |
| KM62 | UGT | Female | Adult | BS | pap | NAD | negative | Negative | 111 | 10.9 | 4.48 | 0.92 | 122 |
| KM63 | Penile | Male | SA | BS | N/A | NAD | negative | Negative | 109 | 8.8 | 2.89 | 0.85 | 100 |
| KM65 | UGT | Female | Adult | BS | back | NAD | negative | Negative | 55 | 8.7 | 2.51 | 0.68 | 57 |
| KM67 | UGT | Female | SA | NBS | no-young | RD | positive | High | 105 | 9.2 | 1.82 | 0.58 | 106 |
| KM68 | UGT | Female | Adult | NBS | pouch | NAD | negative | Negative | 122 | 10.6 | 2.58 | 0.63 | 194 |
| KM69 | UGT | Female | SA | NBS | no-young | RD | positive | Low | 112 | 6.8 | 1.04 | 0.36 | 101 |
| KM70 | UGT | Female | Senior | NBS | no-young | RD | negative | Negative | 53 | 6.1 | 1.65 | 0.57 | 55 |
| KM71 | UGT | Female | Adult | NBS | pouch | NAD | negative | Negative | 179 | 9.5 | 2.69 | 0.74 | 174 |
| KM73 | UGT | Female | SA | NBS | no-young | RD | negative | Negative | 266 | 14.8 | 4.32 | 0.90 | 263 |

| | | | | | | | | | | | | | |
|---|---|---|---|---|---|---|---|---|---|---|---|---|---|
| KM74 | Penile | Male | SA | BS | N/A | NAD | negative | Negative | 153 | 12.2 | 2.83 | 0.80 | 176 |
| KM75 | Penile | Male | Adult | NBS | N/A | RD | positive | Low | 105 | 9.7 | 3.08 | 0.85 | 121 |
| KM77 | UGT | Female | Adult | NBS | no-young | NAD | negative | Negative | 178 | 14.7 | 3.01 | 0.79 | 234 |
| KM79 | UGT | Female | Senior | NBS | no-young | NAD | negative | Negative | 119 | 11.8 | 2.30 | 0.64 | 151 |
| KM81 | Penile | Male | Senior | NBS | N/A | NAD | negative | Negative | 116 | 5.9 | 2.94 | 0.84 | 120 |
| KM83 | UGT | Female | SA | NBS | no-young | RD | positive | Low | 135 | 11.3 | 2.71 | 0.71 | 144 |
| KM85 | UGT | Female | Senior | NBS | no-young | RD | negative | Negative | 209 | 17.9 | 4.13 | 0.88 | 222 |
| KM86 | UGT | Female | SA | NBS | no-young | NAD | negative | Negative | 247 | 18.0 | 3.36 | 0.80 | 273 |
| KM88 | UGT | Female | SA | BS | no-young | NAD | negative | Negative | 162 | 8.7 | 2.70 | 0.80 | 196 |
| KM90 | Penile | Male | SA | NBS | N/A | NAD | negative | Negative | 52 | 5.7 | 2.51 | 0.80 | 60 |
| KM92 | Penile | Male | Senior | NBS | N/A | RD | negative | Negative | 266 | 7.6 | 3.00 | 0.85 | 194 |
| KM93 | Ocular | Male | Senior | NBS | N/A | NAD | negative | negative | 244 | 18.3 | 4.48 | 0.88 | 274 |
| KM95 | UGT | Female | Adult | NBS | pouch | RD | positive | High | 84 | 9.9 | 2.26 | 0.70 | 92 |
| KM99 | UGT | Female | Adult | BS | no-young | NAD | negative | Negative | 59 | 8.8 | 2.23 | 0.64 | 84 |

NB: BS = Breeding season, NBS = non breeding season, SA = sub-adult, SAWJ = sub- adult with joey

**Table S6: List of primers used for Koala microbiome study.**

| Name | Index (5' to 3') | Full primer sequence (5' to 3') | **Source** |
|---|---|---|---|
| 319F_01 | TGCAGATCCAAC | CAAGCAGAAGACGGCATACGAGATGTGACTGGAGTTCAGACGTGTGCTCTTCCGATCTTGCAGATCCAACACTCCTACGGGAGGCAGCAG | 1 |
| 319F_02 | CCATCACATAGG | CAAGCAGAAGACGGCATACGAGATGTGACTGGAGTTCAGACGTGTGCTCTTCCGATCTCCATCACATAGGACTCCTACGGGAGGCAGCAG | 1 |
| 319F_03 | GTGGTATGGGAG | CAAGCAGAAGACGGCATACGAGATGTGACTGGAGTTCAGACGTGTGCTCTTCCGATCTGTGGTATGGGAGTACTCCTACGGGAGGCAGCAG | 1 |
| 319F_04 | ACTTTAAGGGTG | CAAGCAGAAGACGGCATACGAGATGTGACTGGAGTTCAGACGTGTGCTCTTCCGATCTACTTTAAGGGTGTACTCCTACGGGAGGCAGCAG | 1 |
| 319F_05 | GAGCAACATCCT | CAAGCAGAAGACGGCATACGAGATGTGACTGGAGTTCAGACGTGTGCTCTTCCGATCTGAGCAACATCCTTACTCCTACGGGAGGCAGCAG | 1 |
| 319F_06 | TGTTGCGTTTCT | CAAGCAGAAGACGGCATACGAGATGTGACTGGAGTTCAGACGTGTGCTCTTCCGATCTTGTTGCGTTTCTGTACTCCTACGGGAGGCAGCAG | 1 |
| 319F_07 | AGGTACGCAATT | CAAGCAGAAGACGGCATACGAGATGTGACTGGAGTTCAGACGTGTGCTCTTCCGATCTAGGTACGCAATTGTACTCCTACGGGAGGCAGCAG | 1 |
| 319F_08 | ACAGCCACCCAT | CAAGCAGAAGACGGCATACGAGATGTGACTGGAGTTCAGACGTGTGCTCTTCCGATCTACAGCCACCCATCGAACTCCTACGGGAGGCAGCAG | 1 |
| 319F_09 | TGTCTCGCAAGC | CAAGCAGAAGACGGCATACGAGATGTGACTGGAGTTCAGACGTGTGCTCTTCCGATCTTGTCTCGCAAGCCGAACTCCTACGGGAGGCAGCAG | 1 |
| 319F_10 | GAGGAGTAAAGC | CAAGCAGAAGACGGCATACGAGATGTGACTGGAGTTCAGACGTGTGCTCTTCCGATCTGAGGAGTAAAGCCGAACTCCTACGGGAGGCAGCAG | 1 |
| 319F_11 | TACCGCCTCGGA | CAAGCAGAAGACGGCATACGAGATGTGACTGGAGTTCAGACGTGTGCTCTTCCGATCTTACCGCCTCGGAATGAACTCCTACGGGAGGCAGCAG | 1 |
| 319F_12 | CGTAAGATGCCT | CAAGCAGAAGACGGCATACGAGATGTGACTGGAGTTCAGACGTGTGCTCTTCCGATCTCGTAAGATGCCTATGAACTCCTACGGGAGGCAGCAG | 1 |
| 319F_13 | TACCGGCTTGCA | CAAGCAGAAGACGGCATACGAGATGTGACTGGAGTTCAGACGTGTGCTCTTCCGATCTTACCGGCTTGCATGCGAACTCCTACGGGAGGCAGCAG | 1 |
| 319F_14 | ATCTAGTGGCAA | CAAGCAGAAGACGGCATACGAGATGTGACTGGAGTTCAGACGTGTGCTCTTCCGATCTATCTAGTGGCAATGCGAACTCCTACGGGAGGCAGCAG | 1 |
| 319F_15 | CCAGGGACTTCT | CAAGCAGAAGACGGCATACGAGATGTGACTGGAGTTCAGACGTGTGCTCTTCCGATCTCCAGGGACTTCTTGCGTACTCCTACGGGAGGCAGCAG | 1 |
| 319F_16 | CACCTTACCTTA | CAAGCAGAAGACGGCATACGAGATGTGACTGGAGTTCAGACGTGTGCTCTTCCGATCTCACCTTACCTTAGAGTGGACTCCTACGGGAGGCAGCAG | 1 |
| 319F_17 | ATAGTTAGGGCT | CAAGCAGAAGACGGCATACGAGATGTGACTGGAGTTCAGACGTGTGCTCTTCCGATCTATAGTTAGGGCTGAGTGGACTCCTACGGGAGGCAGCAG | 1 |
| 319F_18 | TTAACTGGAAGC | CAAGCAGAAGACGGCATACGAGATGTGACTGGAGTTCAGACGTGTGCTCTTCCGATCTTTAACTGGAAGCCCTGTGGACTCCTACGGGAGGCAGCAG | 1 |
| 319F_19 | CGCGGTTACTAA | CAAGCAGAAGACGGCATACGAGATGTGACTGGAGTTCAGACGTGTGCTCTTCCGATCTCGCGGTTACTAACCTGGAGACTCCTACGGGAGGCAGCAG | 1 |
| 319F_20 | GAGACTATATGC | CAAGCAGAAGACGGCATACGAGATGTGACTGGAGTTCAGACGTGTGCTCTTCCGATCTGAGACTATATGCCCTGGAGACTCCTACGGGAGGCAGCAG | 1 |
| 319F_21 | CCTAAACTACGG | CAAGCAGAAGACGGCATACGAGATGTGACTGGAGTTCAGACGTGTGCTCTTCCGATCTCCTAAACTACGGACTCCTACGGGAGGCAGCAG | 1 |
| 319F_22 | ATGTCCGACCAA | CAAGCAGAAGACGGCATACGAGATGTGACTGGAGTTCAGACGTGTGCTCTTCCGATCTATGTCCGACCAAGTACTCCTACGGGAGGCAGCAG | 1 |
| 319F_23 | GTTACGTGGTTG | CAAGCAGAAGACGGCATACGAGATGTGACTGGAGTTCAGACGTGTGCTCTTCCGATCTGTTACGTGGTTGATGAACTCCTACGGGAGGCAGCAG | 1 |
| 319F_24 | GCACTTCATTTC | CAAGCAGAAGACGGCATACGAGATGTGACTGGAGTTCAGACGTGTGCTCTTCCGATCTGCACTTCATTTCGAGTGGACTCCTACGGGAGGCAGCAG | 1 |
| 806R_01 | CCTAAACTACGG | AATGATACGGCGACCACCGAGATCTACACTCTTTCCCTACACGACGCTCTTCCGATCTCCTAAACTACGGGGACTACHVGGGTWTCTAAT | 1 |
| 806R_02 | TGCAGATCCAAC | AATGATACGGCGACCACCGAGATCTACACTCTTTCCCTACACGACGCTCTTCCGATCTTGCAGATCCAACGGACTACHVGGGTWTCTAAT | 1 |
| 806R_03 | CCATCACATAGG | AATGATACGGCGACCACCGAGATCTACACTCTTTCCCTACACGACGCTCTTCCGATCTCCATCACATAGGGGACTACHVGGGTWTCTAAT | 1 |
| 806R_04 | ACTTTAAGGGTG | AATGATACGGCGACCACCGAGATCTACACTCTTTCCCTACACGACGCTCTTCCGATCTACTTTAAGGGTGAGGACTACHVGGGTWTCTAAT | 1 |
| 806R_05 | GAGCAACATCCT | AATGATACGGCGACCACCGAGATCTACACTCTTTCCCTACACGACGCTCTTCCGATCTGAGCAACATCCTAGGACTACHVGGGTWTCTAAT | 1 |
| 806R_06 | TGTTGCGTTTCT | AATGATACGGCGACCACCGAGATCTACACTCTTTCCCTACACGACGCTCTTCCGATCTTGTTGCGTTTCTTCGGACTACHVGGGTWTCTAAT | 1 |
| 806R_07 | ATGTCCGACCAA | AATGATACGGCGACCACCGAGATCTACACTCTTTCCCTACACGACGCTCTTCCGATCTATGTCCGACCAATCGGACTACHVGGGTWTCTAAT | 1 |
| 806R_08 | AGGTACGCAATT | AATGATACGGCGACCACCGAGATCTACACTCTTTCCCTACACGACGCTCTTCCGATCTAGGTACGCAATTTCGGACTACHVGGGTWTCTAAT | 1 |
| 806R_09 | TGTCTCGCAAGC | AATGATACGGCGACCACCGAGATCTACACTCTTTCCCTACACGACGCTCTTCCGATCTTGTCTCGCAAGCCTAGGACTACHVGGGTWTCTAAT | 1 |
| 806R_10 | GAGGAGTAAAGC | AATGATACGGCGACCACCGAGATCTACACTCTTTCCCTACACGACGCTCTTCCGATCTGAGGAGTAAAGCCTAGGACTACHVGGGTWTCTAAT | 1 |

| | | | |
|---|---|---|---|
| 806R_11 | GTTACGTGGTTG | AATGATACGGCGACCACCGAGATCTACACTCTTTCCCTACACGACGCTCTTCCGATCTGTTACGTGGTTGGATAGGACTACHVGGGTWTCTAAT | 1 |
| 806R_12 | TACCGCCTCGGA | AATGATACGGCGACCACCGAGATCTACACTCTTTCCCTACACGACGCTCTTCCGATCTTACCGCCTCGGAGATAGGACTACHVGGGTWTCTAAT | 1 |
| 806R_13 | CGTAAGATGCCT | AATGATACGGCGACCACCGAGATCTACACTCTTTCCCTACACGACGCTCTTCCGATCTCGTAAGATGCCTGATAGGACTACHVGGGTWTCTAAT | 1 |
| 806R_14 | TACCGGCTTGCA | AATGATACGGCGACCACCGAGATCTACACTCTTTCCCTACACGACGCTCTTCCGATCTTACCGGCTTGCAACTCAGGACTACHVGGGTWTCTAAT | 1 |
| 806R_15 | ATCTAGTGGCAA | AATGATACGGCGACCACCGAGATCTACACTCTTTCCCTACACGACGCTCTTCCGATCTATCTAGTGGCAAACTCAGGACTACHVGGGTWTCTAAT | 1 |
| 806R_16 | CACCTTACCTTA | AATGATACGGCGACCACCGAGATCTACACTCTTTCCCTACACGACGCTCTTCCGATCTCACCTTACCTTATTCTCTGGACTACHVGGGTWTCTAAT | 1 |
| 806R_17 | ATAGTTAGGGCT | AATGATACGGCGACCACCGAGATCTACACTCTTTCCCTACACGACGCTCTTCCGATCTATAGTTAGGGCTTTCTCTGGACTACHVGGGTWTCTAAT | 1 |
| 806R_18 | GCACTTCATTTC | AATGATACGGCGACCACCGAGATCTACACTCTTTCCCTACACGACGCTCTTCCGATCTGCACTTCATTTCTTCTCTGGACTACHVGGGTWTCTAAT | 1 |
| 806R_19 | CGCGGTTACTAA | AATGATACGGCGACCACCGAGATCTACACTCTTTCCCTACACGACGCTCTTCCGATCTCGCGGTTACTAACACTTCTGGACTACHVGGGTWTCTAAT | 1 |
| 806R_20 | GAGACTATATGC | AATGATACGGCGACCACCGAGATCTACACTCTTTCCCTACACGACGCTCTTCCGATCTGAGACTATATGCCACTTCTGGACTACHVGGGTWTCTAAT | 1 |
| 806R_21 | GTGGTATGGGAG | AATGATACGGCGACCACCGAGATCTACACTCTTTCCCTACACGACGCTCTTCCGATCTGTGGTATGGGAGAGGACTACHVGGGTWTCTAAT | 1 |
| 806R_22 | ACAGCCACCCAT | AATGATACGGCGACCACCGAGATCTACACTCTTTCCCTACACGACGCTCTTCCGATCTACAGCCACCCATCTAGGACTACHVGGGTWTCTAAT | 1 |
| 806R_23 | CCAGGGACTTCT | AATGATACGGCGACCACCGAGATCTACACTCTTTCCCTACACGACGCTCTTCCGATCTCCAGGGACTTCTACTCAGGACTACHVGGGTWTCTAAT | 1 |
| 806R_24 | TTAACTGGAAGC | AATGATACGGCGACCACCGAGATCTACACTCTTTCCCTACACGACGCTCTTCCGATCTTTAACTGGAAGCCACTTCTGGACTACHVGGGTWTCTAAT | 1 |
| RTPec.sp- | | AGTCGAACGGAATAATGGCT | 2 |
| RTPec.sp- | | CCAACAAGCTGATATCCCAC | 2 |

[1]Fadrosh DW, Ma B, Gajer P, Sengamalay N, Ott S, Brotman RM, Ravel J: An improved dual-indexing approach for multiplexed 16S rRNA gene sequencing on the Illumina MiSeq platform. Microbiome 2:6 (2014).

[2]Marsh J, Kollipara A, Timms P, & Polkinghorne, A: Novel molecular markers of Chlamydia pecorum genetic diversity in the koala (Phascolarctos cinereus). BMC Microbiology 11, 1-15 (2011).

**Table S7: Taxonomic description for the core OTUs (present in >25% of samples) for koala penile and UGT samples with abbreviations utilised in the manuscript**

| Taxonomy | Abbreviation | UGT | Penile |
|---|---|---|---|
| k__Bacteria;p__Firmicutes;c__Bacilli;o__Lactobacillales;f__Aerococcaceae;g__;s__ | Aerococcaceae_OTU31 | YES | YES |
| k__Bacteria;p__Firmicutes;c__Bacilli;o__Lactobacillales;f__Aerococcaceae;g__Aerococcus;s__ | Aerococcus_OTU1 | YES | YES |
| k__Bacteria;p__Firmicutes;c__Bacilli;o__Lactobacillales;f__Aerococcaceae;g__Aerococcus;s__ | Aerococcus_OTU19 | YES | YES |
| k__Bacteria;p__Firmicutes;c__Bacilli;o__Lactobacillales;f__Aerococcaceae;g__Aerococcus;s__ | Aerococcus_OTU207 | NO | YES |
| k__Bacteria;p__Firmicutes;c__Bacilli;o__Lactobacillales;f__Aerococcaceae;g__Aerococcus;s__ | Aerococcus_OTU208 | NO | YES |
| k__Bacteria;p__Firmicutes;c__Bacilli;o__Lactobacillales;f__Aerococcaceae;g__Aerococcus;s__ | Aerococcus_OTU3 | YES | YES |
| k__Bacteria;p__Firmicutes;c__Bacilli;o__Lactobacillales;f__Aerococcaceae;g__Aerococcus;s__ | Aerococcus_OTU67 | NO | YES |
| k__Bacteria;p__Bacteroidetes;c__Bacteroidia;o__Bacteroidales;f__Bacteroidaceae;g__Bacteroides;s__ | Bacteroides_OTU7 | YES | YES |
| k__Bacteria;p__Proteobacteria;c__Deltaproteobacteria;o__Desulfovibrionales;f__Desulfovibrionaceae;g__Bilophila;s__ | Bilophila | YES | YES |
| k__Bacteria;p__Actinobacteria;c__Actinobacteria;o__Actinomycetales;f__Corynebacteriaceae;g__Corynebacterium;s__ | Boyliae_praeputiale | YES | YES |
| k__Bacteria;p__Proteobacteria;c__Epsilonproteobacteria;o__Campylobacterales;f__Campylobacteraceae;g__Campylobacter;s__ | Campylobacter | YES | YES |
| k__Bacteria;p__Firmicutes;c__Clostridia;o__Clostridiales;f__Clostridiaceae;g__Clostridium;s__ | Clostridium | YES | YES |
| k__Bacteria;p__Actinobacteria;c__Coriobacteriia;o__Coriobacteriales;f__Coriobacteriaceae;g__;s__Corynebacterium | Coriobacteriaceae | YES | YES |
| k__Bacteria;p__Actinobacteria;c__Coriobacteriia;o__Coriobacteriales;f__Coriobacteriaceae;g__;s__Corynebacterium | Corynebacterium_OTU325 | NO | YES |
| k__Bacteria;p__Actinobacteria;c__Coriobacteriia;o__Coriobacteriales;f__Coriobacteriaceae;g__;s__Corynebacterium | Corynebacterium_OTU368 | NO | YES |
| k__Bacteria;p__Actinobacteria;c__Coriobacteriia;o__Coriobacteriales;f__Coriobacteriaceae;g__;s__Corynebacterium | Corynebacterium_OTU390 | NO | YES |
| k__Bacteria;p__Proteobacteria;c__Deltaproteobacteria;o__Desulfarculales;f__Desulfarculaceae;g__;s__ | Desulfarculaceae_OTU170 | YES | YES |
| k__Bacteria;p__Proteobacteria;c__Gammaproteobacteria;o__Enterobacteriales;f__Enterobacteriaceae;g__;s__ | Enterobacteriaceae_OTU29 | YES | YES |
| k__Bacteria;p__Firmicutes;c__Bacilli;o__Lactobacillales;f__Aerococcaceae;g__Facklamia;s__ | Facklamia | YES | YES |
| k__Bacteria;p__Bacteroidetes;c__Flavobacteriia;o__Flavobacteriales;f__Flavobacteriaceae;g__Flavobacterium;s__ | Flavobacterium | YES | YES |
| k__Bacteria;p__Firmicutes;c__Clostridia;o__Clostridiales;f__Lachnospiraceae;g__;s__ | Lachnospiraceae_OTU10 | YES | YES |
| k__Bacteria;p__Firmicutes;c__Clostridia;o__Clostridiales;f__Ruminococcaceae;g__Oscillospira;s__ | Oscillospira_OTU871 | YES | YES |
| k__Bacteria;p__Firmicutes;c__Clostridia;o__Clostridiales;f__[Tissierellaceae];g__Peptoniphilus;s__ | Peptoniphilus_OTU50 | NO | YES |
| k__Bacteria;p__Firmicutes;c__Clostridia;o__Clostridiales;f__[Tissierellaceae];g__Peptoniphilus;s__ | Peptoniphilus_OTU66 | YES | YES |
| k__Bacteria;p__Firmicutes;c__Clostridia;o__Clostridiales;f__Veillonellaceae;g__Phascolarctobacterium;s__ | Phascolarctobacterium | YES | YES |
| k__Bacteria;p__Bacteroidetes;c__Bacteroidia;o__Bacteroidales;f__Porphyromonadaceae;g__Porphyromonas;s__ | Porphyromonas | NO | YES |
| k__Bacteria;p__Actinobacteria;c__Actinobacteria;o__Actinomycetales;f__Propionibacteriaceae;g__;s__ | Propionibacteriaceae_OTU0 | YES | YES |
| k__Bacteria;p__Actinobacteria;c__Actinobacteria;o__Actinomycetales;f__Propionibacteriaceae;g__;s__ | Propionibacteriaceae_OTU4 | YES | YES |
| k__Bacteria;p__Actinobacteria;c__Actinobacteria;o__Actinomycetales;f__Propionibacteriaceae;g__;s__ | Propionibacteriaceae_OTU64 | NO | YES |
| k__Bacteria;p__Actinobacteria;c__Actinobacteria;o__Actinomycetales;f__Propionibacteriaceae;g__Propionibacterium;s__acnes | Propionibacterium_acnes | YES | YES |
| k__Bacteria; p__TM7; c__TM7-3; o__I025; f__Rs-045; g__; s__ | Rs-045 | NO | YES |
| k__Bacteria;p__Synergistetes;c__Synergistia;o__Synergistales;f__Synergistaceae;g__;s__ | Synergistaceae | YES | YES |
| k__Bacteria;p__Actinobacteria;c__Actinobacteria;o__Actinomycetales;f__Actinomycetaceae;g__Trueperella;s__ | Trueperella | NO | YES |
| k__Bacteria;p__Proteobacteria;c__Alphaproteobacteria;o__Rhizobiales;f__Rhizobiaceae;g__Agrobacterium;s__ | Agrobacterium | YES | NO |

| | | | |
|---|---|---|---|
| k__Bacteria;p__Bacteroidetes;c__Bacteroidia;o__Bacteroidales;f__Bacteroidaceae;g__Bacteroides;s__fragilis | Bacteroides_fragilis | YES | NO |
| k__Bacteria;p__Bacteroidetes;c__Bacteroidia;o__Bacteroidales;f__Bacteroidaceae;g__Bacteroides;s__ | Bacteroides_OTU21 | YES | NO |
| k__Bacteria;p__Actinobacteria;c__Actinobacteria;o__Actinomycetales;f__Microbacteriaceae;g__CandidatusRhodoluna;s__ | Ca_Rhodoluna | YES | NO |
| k__Bacteria;p__Firmicutes;c__Clostridia;o__Clostridiales;f__;g__;s__ | Clostridiales_OTU22 | YES | NO |
| k__Bacteria;p__Firmicutes;c__Clostridia;o__Clostridiales;f__;g__;s__ | Clostridiales_OTU32 | YES | NO |
| k__Bacteria;p__Bacteroidetes;c__Cytophagia;o__Cytophagales;f__Cytophagaceae;g__;s__ | Cytophagaceae | YES | NO |
| k__Bacteria;p__Proteobacteria;c__Betaproteobacteria;o__Burkholderiales;f__Comamonadaceae;g__Delftia;s__ | Delftia | YES | NO |
| k__Bacteria;p__Proteobacteria;c__Deltaproteobacteria;o__Desulfarculales;f__Desulfarculaceae;g__;s__ | Desulfarculaceae_OTU286 | YES | NO |
| k__Bacteria;p__Proteobacteria;c__Deltaproteobacteria;o__Desulfovibrionales;f__Desulfovibrionaceae;g__;s__ | Desulfovibrionaceae | YES | NO |
| k__Bacteria;p__Proteobacteria;c__Gammaproteobacteria;o__Enterobacteriales;f__Enterobacteriaceae;g__;s__ | Enterobacteriaceae_OTU209 | YES | NO |
| k__Bacteria;p__Proteobacteria;c__Gammaproteobacteria;o__Enterobacteriales;f__Enterobacteriaceae;g__;s__ | Enterobacteriaceae_OTU731 | YES | NO |
| k__Bacteria;p__Proteobacteria;c__Gammaproteobacteria;o__Enterobacteriales;f__Enterobacteriaceae;g__;s__ | Enterobacteriaceae_OTU868 | YES | NO |
| k__Bacteria;p__Fusobacteria;c__Fusobacteriia;o__Fusobacteriales;f__Fusobacteriaceae;g__Fusobacterium;s__ | Fusobacterium | YES | NO |
| k__Bacteria;p__TM7;c__TM7-3;o__I025;f__Rs-045;g__;s__ | I025 | YES | NO |
| k__Bacteria;p__Firmicutes;c__Clostridia;o__Clostridiales;f__Lachnospiraceae;g__;s__ | Lachnospiraceae_OTU11 | YES | NO |
| k__Bacteria;p__Firmicutes;c__Clostridia;o__Clostridiales;f__Lachnospiraceae;g__;s__ | Lachnospiraceae_OTU14 | YES | NO |
| k__Bacteria;p__Firmicutes;c__Clostridia;o__Clostridiales;f__Lachnospiraceae;g__;s__ | Lachnospiraceae_OTU37 | YES | NO |
| k__Bacteria;p__Firmicutes;c__Clostridia;o__Clostridiales;f__Lachnospiraceae;g__;s__ | Lachnospiraceae_OTU726 | YES | NO |
| k__Bacteria;p__Proteobacteria;c__Gammaproteobacteria;o__Pasteurellales;f__Pasteurellaceae;g__Lonepinella;s__koalarum | Lonepinella koalarum | YES | NO |
| k__Bacteria;p__Firmicutes;c__Clostridia;o__Clostridiales;f__Ruminococcaceae;g__Oscillospira;s__ | Oscillospira_OTU143 | YES | NO |
| k__Bacteria;p__Firmicutes;c__Clostridia;o__Clostridiales;f__Ruminococcaceae;g__Oscillospira;s__ | Oscillospira_OTU69 | YES | NO |
| k__Bacteria;p__Firmicutes;c__Clostridia;o__Clostridiales;f__Ruminococcaceae;g__Oscillospira;s__ | Oscillospira_OTU855 | YES | NO |
| k__Bacteria;p__Proteobacteria;c__Betaproteobacteria;o__Burkholderiales;f__Oxalobacteraceae;g__Oxalobacter;s__formigenes | Oxalobacter_formigene | YES | NO |
| k__Bacteria;p__Bacteroidetes;c__Bacteroidia;o__Bacteroidales;f__Porphyromonadaceae;g__Parabacteroides;s__distasonis | Parabacteroides_distasonis | YES | NO |
| k__Bacteria;p__Bacteroidetes;c__Bacteroidia;o__Bacteroidales;f__Porphyromonadaceae;g__Parabacteroides;s__ | Parabacteroides_OTU18 | YES | NO |
| k__Bacteria;p__Bacteroidetes;c__Bacteroidia;o__Bacteroidales;f__Porphyromonadaceae;g__Parabacteroides;s__ | Parabacteroides_OTU24 | YES | NO |
| k__Bacteria;p__Bacteroidetes;c__Bacteroidia;o__Bacteroidales;f__Porphyromonadaceae;g__Parabacteroides;s__ | Parabacteroides_OTU266 | YES | NO |
| k__Bacteria;p__Bacteroidetes;c__Bacteroidia;o__Bacteroidales;f__Porphyromonadaceae;g__Parabacteroides;s__ | Parabacteroides_OTU375 | YES | NO |
| k__Bacteria;p__Planctomycetes;c__vadinHA49;o__PeHg47;f__;g__;s__ | PeHg47_OTU27 | YES | NO |
| k__Bacteria;p__Planctomycetes;c__vadinHA49;o__PeHg47;f__;g__;s__ | PeHg47_OTU766 | YES | NO |
| k__Bacteria;p__Planctomycetes;c__vadinHA49;o__PeHg47;f__;g__;s__ | PeHg47_OTU86 | YES | NO |
| k__Bacteria;p__Bacteroidetes;c__Bacteroidia;o__Bacteroidales;f__Rikenellaceae;g__;s__ | Rikenellaceae | YES | NO |
| k__Bacteria;p__Firmicutes;c__Clostridia;o__Clostridiales;f__Ruminococcaceae;g__;s__ | Ruminococcaceae_OTU16 | YES | NO |
| k__Bacteria;p__Firmicutes;c__Clostridia;o__Clostridiales;f__Ruminococcaceae;g__;s__ | Ruminococcaceae_OTU23 | YES | NO |
| k__Bacteria;p__Firmicutes;c__Clostridia;o__Clostridiales;f__Ruminococcaceae;g__;s__ | Ruminococcaceae_OTU267 | YES | NO |
| k__Bacteria;p__Firmicutes;c__Clostridia;o__Clostridiales;f__Ruminococcaceae;g__;s__ | Ruminococcaceae_OTU365 | YES | NO |

| | | | |
|---|---|---|---|
| k__Bacteria;p__Firmicutes;c__Clostridia;o__Clostridiales;f__Ruminococcaceae;g__;s__ | Ruminococcaceae_OTU589 | YES | NO |
| k__Bacteria;p__Firmicutes;c__Clostridia;o__Clostridiales;f__Ruminococcaceae;g__;s__ | Ruminococcaceae_OTU629 | YES | NO |
| k__Bacteria;p__Firmicutes;c__Clostridia;o__Clostridiales;f__Ruminococcaceae;g__;s__ | Ruminococcaceae_OTU661 | YES | NO |
| k__Bacteria;p__Firmicutes;c__Clostridia;o__Clostridiales;f__Ruminococcaceae;g__Ruminococcus;s__ | Ruminococcus_OTU26 | YES | NO |
| k__Bacteria;p__Firmicutes;c__Clostridia;o__Clostridiales;f__Ruminococcaceae;g__Ruminococcus;s__ | Ruminococcus_OTU449 | YES | NO |
| k__Bacteria;p__Firmicutes;c__Bacilli;o__Lactobacillales;f__Streptococcaceae;g__Streptococcus;s__ | Streptococcus | YES | NO |
| k__Bacteria;p__Synergistetes;c__Synergistia;o__Synergistales;f__Synergistaceae;g__vadinCA02;s__ | vadinCA02 | YES | NO |
| k__Bacteria;p__Cyanobacteria;c__4C0d-2;o__YS2;f__;g__;s__ | YS2 | YES | NO |
